# Genome-wide association analysis reveal candidate genes and haplotypes related to root weight in cucumber (*Cucumis sativus* L.)

**DOI:** 10.3389/fpls.2024.1417314

**Published:** 2024-07-17

**Authors:** Zhuonan Dai, Shaoyun Dong, Hexu Cai, Diane M. Beckles, Jiantao Guan, Xiaoping Liu, Xingfang Gu, Han Miao, Shengping Zhang

**Affiliations:** ^1^ State Key Laboratory of Vegetable Biobreeding, Institute of Vegetables and Flowers, Chinese Academy of Agricultural Sciences, Beijing, China; ^2^ Department of Plant Sciences, University of California, Davis, Davis, CA, United States

**Keywords:** cucumber (*Cucumis sativus* L.), root, GWAS (genome-wide association study), candidate genes, haplotypes

## Abstract

**Background:**

The plant root system is critical for the absorption of water and nutrients, and have a direct influence on growth and yield. In cucumber, a globally consumed crop, the molecular mechanism of root development remains unclear, and this has implications for developing stress tolerant varieties. This study sought to determine the genetic patterns and related genes of cucumber root weight. A core cucumber germplasms population was used to do the GWAS analysis in three environments.

**Results:**

Here, we investigated four root-weight related traits including root fresh weight (RFW), root dry weight (RDW), ratio of root dry weight to root fresh weight (RDFW) and the comprehensive evaluation index, D-value of root weight (DRW) deduced based on the above three traits for the core germplasm of the cucumber global repository. According to the D-value, we identified 21 and 16 accessions with light and heavy-root, respectively. We also found that the East Asian ecotype accessions had significantly heavier root than other three ecotypes. The genome-wide association study (GWAS) for these four traits reveals that 4 of 10 significant loci (gDRW3.1, gDRW3.2, gDRW4.1 and gDRW5.1) were repeatedly detected for at least two traits. Further haplotype and expression analysis for protein-coding genes positioned within these 4 loci between light and heavy-root accessions predicted five candidate genes (i.e., Csa3G132020 and Csa3G132520 both encoding F-box protein PP2-B1 for gDRW3.1, Csa3G629240 encoding a B-cell receptor-associated protein for gDRW3.2, Csa4G499330 encodes a GTP binding protein for gDRW4.1, and Csa5G286040 encodes a proteinase inhibitor for gDRW5.1).

**Conclusions:**

We conducted a systematic analysis of the root genetic basis and characteristics of cucumber core germplasms population. We detected four novel loci, which regulate the root weight in cucumber. Our study provides valuable candidate genes and haplotypes for the improvement of root system in cucumber breeding.

## 1 Introduction

Cucumber belongs to the Cucurbitaceae and is an important vegetable crop in the world. In 2021, the harvested area of cucumber reached 3,464,737 hectares, producing 169,126,456 tons worldwide (FAOSTAT, 2021). However, cucumber has a shallow root system that weakly absorbs water and fertilizer (Beyaert et al., 2007). It is usually necessary to increase cucumber yield through grafting (Huang et al., 2009; Li et al., 2014; Usanmaz and Abak, 2019). In recent years, breeders have made efforts to further improve crop yield through root improvement (Dorlodot et al., 2007; Uga et al., 2013). Some studies have examined the root architecture and branching patterns in cucumber (Zhang et al., 2012; Kiryushkin et al., 2024; Cai et al., 2021). It is known that cucumbers have longer taproots and shallower fibrous roots. The lateral roots branching out from the primary root (Mao et al., 2003). However, the specific genetic factors and regulatory mechanisms controlling root architecture in cucumber are not yet fully characterized. Compared to other crops such as *Oryza sativa* L (Kamoshita et al., 2002; Courtois et al., 2009), *Zea mays* L (Hochholdinger and Tuberosa, 2009; Burton et al., 2015), *Glycine max* (Liang et al., 2014, Liang et al., 2017) and the model plant *Arabidopsis thaliana* (Fukaki et al., 2002; Knight, 2007), there is less research on the genetic diversity and key regulatory genes of cucumber roots, which hinders future genetic breeding.

Root development is influenced by many factors, such as internal and external environments. As one of the endogenous factors, phytohormones [i.e., ethylene, auxin, gibberellin (GA), cytokinin (CK), jasmonic acid (JA), and brassinosteroid (BR)] and their crosstalk have been shown to play vital roles in the regulation of root growth (Li et al., 2022). Auxin, as one of the well-studied phytohormones, plays a critical role in regulating the elongation of primary and lateral roots (Ljung et al., 2005). Himanen et al. (2002) reported that auxin can promote lateral root formation by inhibiting the expression of cell division inhibitors *KRP1* and *KRP2*. BR can also act synergically with auxin, and regulate the polar transport of auxin to promote lateral root development (Bao et al., 2004). Auxin can also disrupt the stability of the DELLA protein by regulating gibberellin (GA), thus affecting the elongation of cells in the root elongation zone (Fu and Harberd, 2003). Ethylene participates in the differentiation of root hair cells and can promote the elongation of root hair (Tanimoto et al., 1995; Pitts et al., 1998). JA plays a positive role in root development. F-box protein COI1 is a JA receptor, which positively regulates JA response. Some studies show that COI1/JAZ mediated JA signaling pathway is involved in the regulation of root hair elongation (Han et al., 2020). CK can regulate the development of root hairs by regulating the expression of C2H2 zinc finger protein ZFP5 (An et al., 2012). Applying exogenous CK can increase the length and density of hair (Zhang et al., 2016; Huang et al., 2020). The proper concentration of GA_3_ treatment had a strong promoting effect on the root dry weight and the root/shoot dry weight ratio of cucumber (Cai et al., 2021). In addition, studies have shown that exogenous ethylene can induce the development of cucumber adventitious roots (Deng et al., 2022).

Multiple regulatory genes involved in root formation have also been previously identified in other species. In *Arabidopsis*, *SCR* and *SHR* can regulate the expression of the auxin influx vector LAX3, and promote the development of primary and lateral roots (Aida et al., 2004). The *PTL* class of transcription factors are responsive to auxin accumulation signals, and co-regulate root stem cell differentiation with auxin response factors (ARFs) (Aida et al., 2004; Ding and Friml, 2010). *Dig6* can regulate lateral root development by affecting the transport and distribution of auxin (Zhao et al., 2015). In rice, the *OsCAND1* gene is important for taproot formation in rice, and controls taproot growth by participating in auxin signaling and maintaining the G2/M cell cycle transition in meristem (Wang et al., 2011). The *SOLITARY-ROOT/IAA14* gene is associated with lateral root development (Fukaki et al., 2002). Members of the CRL family have been found to perform different biological functions in root formation. For example, *CRL1* inhibits adventitious root formation; *CRL2* is related to the formation of taproot and lateral root primordia; *CRL3* is related to the formation of taproot primordia; *CRL4* promotes adventitious root formation; and *CRL5* can be induced by auxin to form taproots (Inukai et al., 2001, Inukai et al., 2005; Liu et al., 2009; Kitomi et al., 2011; Woo et al., 2018). In cucumber, Yan et al. (2018) found that *CsGPA1* controlled root growth by promoting the cell size and meristem of cucumber tip cells through the study of G protein. *CsCEP4* peptide can promote the growth of cucumber primary roots by regulating reactive oxygen species (Liu et al., 2021). The amino acid transporter *CsAAP2* can mediate polar auxin transport in cucumber root tip to influence root development (Yao et al., 2023). However, the key regulators of root growth still remained unknown in cucumber, hindering the exploration of the regulatory network and its further genetic improvement.

In recent years, with the development of resequencing technology, GWAS has been widely used to identify loci that influence plant root traits. Beyer et al. (2019) evaluated five root traits of 211 hexaploid wheat materials at the seedling stage and identified 63 marker-trait associations (MTA). Wang et al. (2019) carried out a genome-wide association mapping of 13 root traits and three aboveground traits with 297 maize inbred lines, and three pleiotropic QTLs involving five root traits were detected. Pace et al. (2015) used 384 maize inbred lines to conduct a genome-wide association analysis on 22 root architecture of seedlings, and significant SNPs for multiple traits were located on gene model *GRMZM2G153722*. Li et al. (2021) measured 280 *Brassica napus* accessions with five consecutive nutrient stages and identified 16 persistent and 32 stage-specific quantitative trait loci (QTLs) through GWAS. Chen et al. (2021) re-sequenced 220 alfalfa core germplasm, and identified 26 loci for fresh root weight, 35 for dry root weight, 3 for root length, and 3 for root number through GWAS. In cucumber, Qi et al. (2013) has re-sequenced a core collection of 115 cucumber accessions that capture 77.2% of the total genetic diversity estimated for 3,342 accessions from a wide geographic distribution, which provided a rich germplasm panel representing the high diversity and suitable for the genetic dissection of root-related traits.

Here, we performed GWAS for four root-weight related traits of 96 core germplasm accessions and identified 10 significant loci. Furthermore, using haplotype and expression analysis between heavy and light-root lines, five candidate genes positioned within four repeatedly detected loci were determined. This study therefore establishes new loci that may be used for breeding new cucumber varieties with novel and robust roots trait, and provides new ideas for elucidating mechanisms related to root development in cucumber.

## 2 Materials and methods

### 2.1 Plant materials

A total of 96 accessions, a representative collection selected from the global 3,342 accessions, were used for the GWAS (**Supplementary Table S1**), which includes all the four geographic groups including East Asian, Eurasian, Indian, and Xishuangbanna (Qi et al., 2013). These materials have been used for genome-wide association analysis of various cucumber traits (Liu et al., 2020, Liu et al., 2021a; Han et al., 2023; Li et al., 2023). These accessions were cultivated at the Institute of Vegetables and Flowers (IVF), Chinese Academy of Agricultural Sciences (CAAS) in three distinct seasons (spring and autumn at 2017 and spring at 2020). Seedlings were grown in a soilless culture with vermiculite as the growing medium. The seeds were sown in a 4×8 seedling tray and irrigated with water. Seven days after sowing, the seedlings were irrigated with 500 mL of the Hoagland working nutrient solution (**Supplementary Table S2**), per seedling plate per day. All the experiments adopted a random block design with three replicates, and five plants planted for each replicate, for each accession. The tested 96 accessions were provided by Cucumber Breeding Group of IVF, CAAS.

### 2.2 Investigation and analysis of phenotypic data

Four root-weight related traits were evaluated for the 96 accessions including root fresh weight (RFW), root dry weight (RDW), root dry weight/root fresh weight (RDFW), and D-value of root weight (DRW). When the seedlings grew with two fully expanded true leaves at 14 days after sowing, the roots were washed with water, and all moisture on the surface of the root was removed with absorbent paper. RFW was determined using an analytical balance. RDW was determined after drying at 60℃ until the root weight was constant (Nayyeripasand et al., 2021). When a trait is evaluated by multiple indicators, a comprehensive evaluation indicator D-value can be introduced through principal component analysis and membership function analysis (Xie et al., 2021). In this study, the D-value of root weight (DRW), as the comprehensive index to evaluate root weight, was computed using the membership function values from the principal component analysis of RFW, RDW and RDFW. Principal component weight W_j_ = I_j_/∑I_j_ (j = 1, 2, …, n), where I_j_ represents the contribution rate of the J^th^ principal component. Membership function U (X_j_) = (X_j_ − X_min_)/(X_max_ −X_min_) (j = 1, 2, …, n), where X_j_ represents the J^th^ principal component value, X_min_ and X_max_ represent the minimum and maximum values of the J^th^ principal component in different strains, respectively. The D-value was calculated as D = ∑ (U_j_ × W_j_) (j = 1, 2, …, n). Therefore, a total of four traits (RFW, RDW, RDFW, DRW) were involved in the evaluation and analysis of root weight (**Supplementary Table S3**).

Best Linear Unbiased Prediction (BLUP) has been reported to effectively integrate multiple environmental data, remove environmental effects, and obtain stable genetic phenotypes of individuals (Cumbie et al., 2011; Liu et al., 2022; Han et al., 2023). Therefore, we used the BLUP values across the three environments for each of the four root-weight related traits. The ‘lme40’ package of R V3.6.1 software (www.r-project.org) was used to calculate BLUP values (Liu et al., 2022). SAS V9.4 software was used for statistical analysis of phenotypic data. TBtools V1.0692 software was used to perform phylogenetic analysis and heat map plotting for the DRW (Chen et al., 2018).

### 2.3 Genome-wide association analysis

Genotypic data of 96 cucumber accessions were obtained from publicly available sequence data NCBI Short Read Archive (SRA) under accession SRA056480 (http://www.ncbi.nlm.nih.gov/sra?term=SRA056480) (Qi et al., 2013). The Fast-LMM (factored spectrally transformed linear mixed model) software was used to perform the GWAS (Lippert et al., 2011) with an estimated relatedness matrix estimated using 1,547,181 SNPs. Compared to other methods, Fast-LMM can capture confounding factors and address confounding factors by using genetic similarity methods (Runcie and Crawford, 2019). In this study, GWAS were performed for both phenotypic data and BLUP data from three seasons. The genome-wide significance threshold was established through Bonferroni correction (i.e., corrected *P* = 1/n, where n represents the number of independent SNPs across the genome), a method frequently applied in various studies (Yang et al., 2014; Huang et al., 2017; Liu et al., 2021b; You et al., 2023). The value of n was determined by SNP dataset pruning with PLINK v1.9 using parameters ‘1000kb 10kb 0.2’, resulting in the retention of 55,152 independent SNPs. Thus, the significance threshold selected was -log_10_ *P* = 5.00. Manhattan plot was generated using R package qqman (Turner, 2014). Plink software was used to analyze the linkage disequilibrium (LD) block and calculate the linkage disequilibrium (LD) decay coefficients (r^2^) among high-density SNPs, and was used to evaluate LD decay (Purcell et al., 2007). An r^2^ ≥ 0.6 was chosen as the threshold to define the LD block (Li et al., 2023). The pairwise linkage disequilibrium heat map was plotted using the LDheatmap package (Shin et al., 2006).

### 2.4 Determination of candidate genes positioned within the significant loci

The LD blocks containing the peak SNP was considered as the candidate region for further analysis. Annotations of the protein-coding genes within the candidate region for each locus were determined using the Chinese Long V2 genome on Cucumber genome website (http://cucurbitgenomics.org/). The analysis of candidate protein coding genes was conducted by using qPCR to analyze the relative expression levels of 4 light root germplasm (‘CG54’, ‘CG77’, ‘CG49’, ‘4795’) and 3 heavy root germplasm (‘CG108’, ‘CG28’, ‘3691’), and the relative expression levels of light-root ‘4795’ and heavy-root ‘3691’ in multiple tissues. The roots of ‘4795’ and ‘3691’ accessions showed differences in mass from the cotyledon stage (**Supplementary Figure S1**). Roots, stems, cotyledons, and the true leaves were collected at the two true leaves flattening stage. Samples were stored at -80°C until further analyzed. Total RNA of samples was extracted using Plant RNA Extraction Kit (TaKaRa MiniBEST), and first-strand cDNA was synthesized using HiScripRIII RT SuperMix for qPCR (Vazyme Biotech). ChamQ Universal SYBR qPCR Master Mix (Vazyme Biotech) was used for qRT-PCR. The expression level of *CsActin1* (*Csa3G806800*) was used as the standardized control, and the 2^−ΔCt^ method was used to calculate gene expression levels (Schmittgen and Zakrajsek, 2000). Three biological replicates and three technical replicates were set up to obtain the expression level of each gene. Primer3.0 was used to design gene-specific primers (https://primer3.ut.ee/). All primer information was listed in **Supplementary Table S4.**

The resequencing data of 16 heavy and 17 light-root accessions excavated by systematic cluster analysis were used for haplotype analysis of each candidate gene. The cis-acting elements at the putative promoter region (< 2.0kb from start codon ATG) were predicted using the PlantCARE tool (http://bioinformatics.psb.ugent.be/webtools/plantcare/html/) (Li et al., 2023).

## 3 Results

### 3.1 Phenotypic variation and clustering analysis of root-weight related traits for cucumber core germplasm

We measured the fresh weight (RFW) and dry weight (RDW) data of roots for the 96 core accessions in three different environments (i.e., spring and autumn of 2017 as well as the spring of 2020) and also computed the RDFW value. In order to comprehensively evaluate the root weight related traits, we calculated the D-value of root weight (DRW) index based on principal component analysis (PCA) and membership function analysis. We also estimated the BLUP values for RFW, RDW, and RDFW to eliminate environmental influences for further analysis. Through PCA of the above three phenotypes (RFW, RDW and RDFW), three new indicators (Prin1, Prin2 and Prin3) were obtained. In this study, Prin1 mainly explained 59.99% RDW, 75.71% RFW, and -25.85% RDFW. Prin2 explained 54.58% RDW, 82.34% RDFW, and -15.21% RFW. Prin1 mainly represents root dry weight and root fresh weight, and Prin2 mainly represents root dry weight and root dry-fresh ratio. The cumulative contribution rate of Prin1 and Prin2 reached up to 95.86%. Thus, Prin1 and Prin2 were used to calculate DRW (**Supplementary Table S4**).

**Figure 1:**
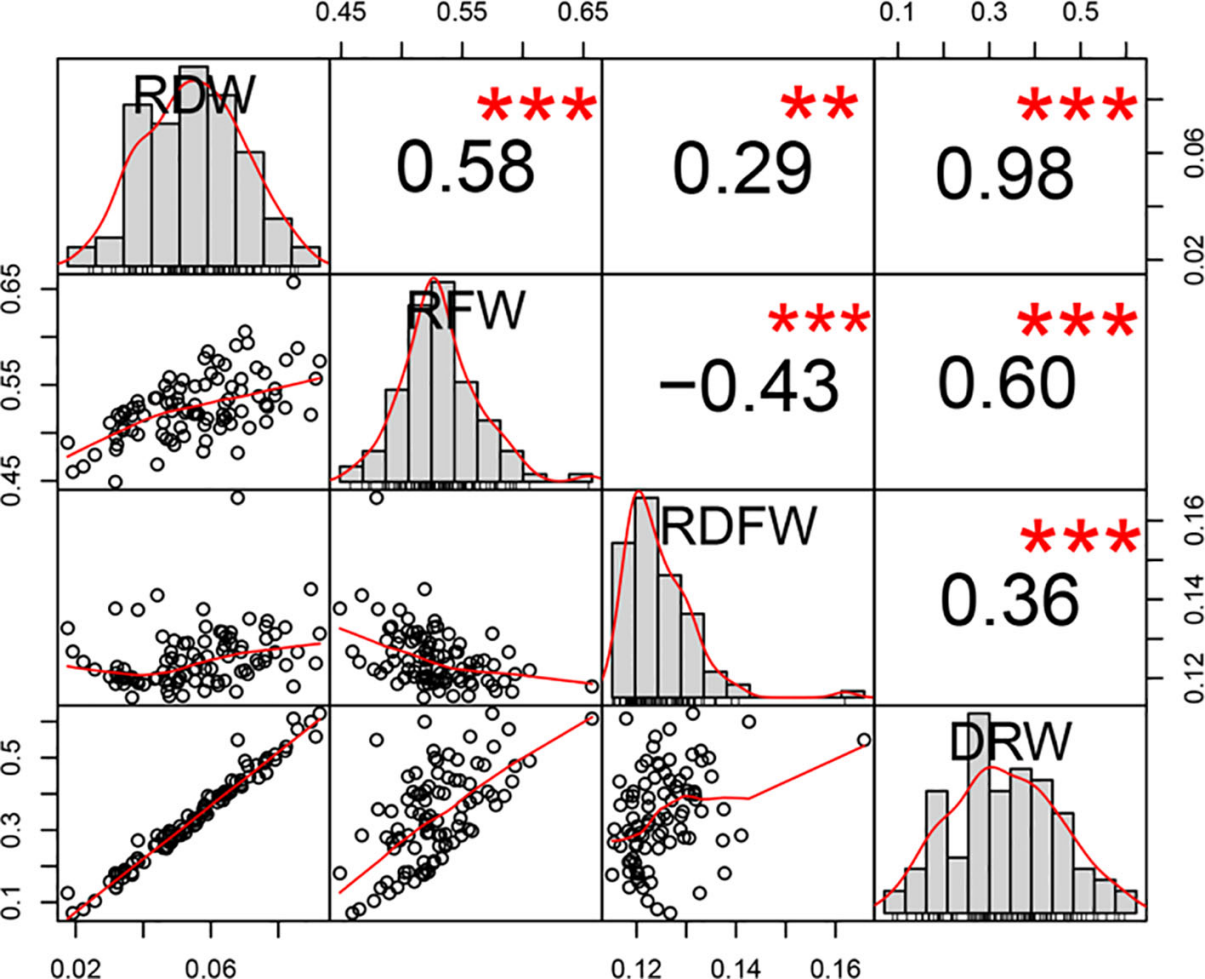
Distribution and Pearson correlation analysis among the four root-weight related traits in 96 cucumber core germplasm. **P < 0.01, ***P < 0.001 using two-sided student’s t-tests.

We next evaluated the phenotypic variation of these four traits. RDW ranged from 0.0176g to 0.0922g (with a mean of 0.0550g), RFW ranged from 0.4490g to 0.6571g (with a mean of 0.5274g), RDFW from 0.1151 to 0.1658 (with a mean of 0.1251), and DRW from 0.0702 to 0.6223 (with a mean of 0.3365). RDW and DRW had higher coefficient of variation (CV) values (31.4288% and 38.0553%, respectively), compared with the other two traits (6.4387% and 5.8995% for RFW and RDFW) (**Table 1**). Pearson correlation analysis between any two of these four traits showed significantly positive correlation (*P* < 0.001 using two-sided student’s t-tests), except for the negative correlation between RFW and RDFW. Also, RDW had the strongest correlation with DRW (r = 0.98) (**Figure 1**).

**Table 1:** Phenotypic variations (Standard deviation, SD; Minimum, Min; Maximum, Max; Coefficient of Variation, CV) of RDW, RFW, RDFW, and DRW.

Traits	Mean	SD	Min	Max	CV (%)
RDW	0.0550	0.0173	0.0176	0.0922	31.4288
RFW	0.5274	0.0340	0.4489	0.6571	6.4387
RDFW	0.1251	0.0074	0.1151	0.1658	5.8953
DRW	0.3365	0.1281	0.0702	0.6223	38.0553

**Figure 2:**
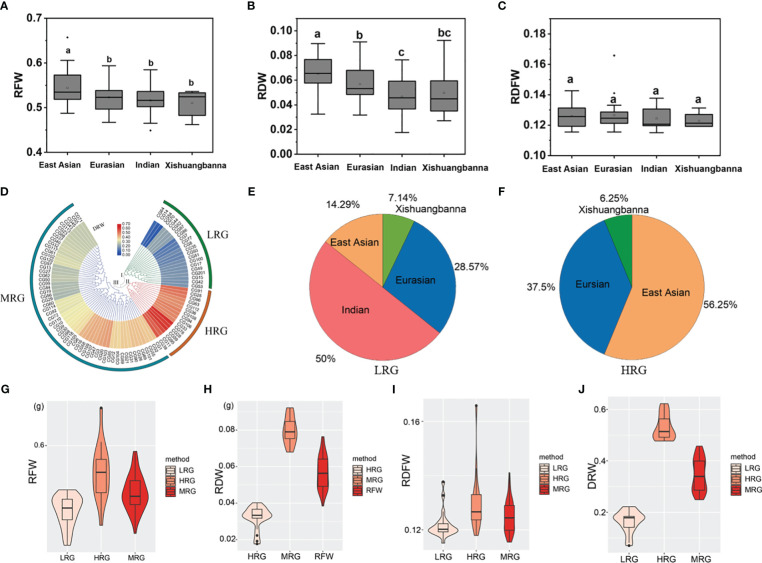
Cluster analysis of D-value of root weight (DRW) data derived from the cucumber core germplasm. **(A–B)** A histogram of RFW, RDW, and RDFW of four main geographic groups. a, b, and c represent the level of significant difference from high to low. **(D)** A heatmap of DRW of the cucumber core germplasm. **(E)** Distribution of four major geographic groups in light-root germplasm (LRG). **(F)** Distribution of four major geographic groups in heavy-root germplasm (HRG). **(G–J)** A histogram of RFW, RDW, RDFW, and DRW of LRG, HRG, and MRG.

According to the geographical source, the 96 core accessions could be classified into four types including East Asian, Eurasian, Indian, and Xishuangbanna (Qi et al., 2013). Comparisons of RFW and RDW between different geographical types showed that the root of the East Asian group was the heaviest among the four geographical types (*P* < 0.05 using two-sided student’s t-tests) (**Figures 2A–C**). To further identify the germplasm exhibiting extremely heavy and light root, we perform clustering analysis using DRW data. The results showed that the core germplasm could be divided into three groups (**Figure 2D**): group I consisting of 21 light-root germplasm (LRG) with the lower mean values of RFW (0.5013 ± 0.0239 g), RDW (0.0321 ± 0.0061 g), RDFW (0.1219 ± 0.0051), and DRW (0.1645 ± 0.0417); group II comprising 16 heavy-root germplasm (HRG) with higher mean values of RFW (0.5593 ± 0.0417 g), RDW (0.0798 ± 0.0075 g), RDFW (0.1301 ± 0.0114), and DRW (0.5309 ± 0.0490). Other 57 medium-root germplasm (MRG) in group III showed intermediate mean values for RFW (0.5280 ± 0.0265 g), RDW (0.0570 ± 0.0091 g), RDFW (0.1249 ± 0.0059), and DRW (0.3453 ± 0.0632) (**Figures 2G–J**). Notably, 50% of LRG in group I were Indian types, whereas, 56.25% of HRG in group II were East Asian types (**Figures 2E, F**). This was consistent with the aforementioned results that East Asian type accessions showed heaviest root weight, but Indian type the lightest root weight. Both results demonstrated that the East Asian type accessions with heavy root may be preferred by local breeders or producers with heavier root and suggest that these materials have huge potential for utilization in improving the root weight traits in the further genetic breeding.

### 3.2 GWAS analysis of root weight

**Figure 3:**
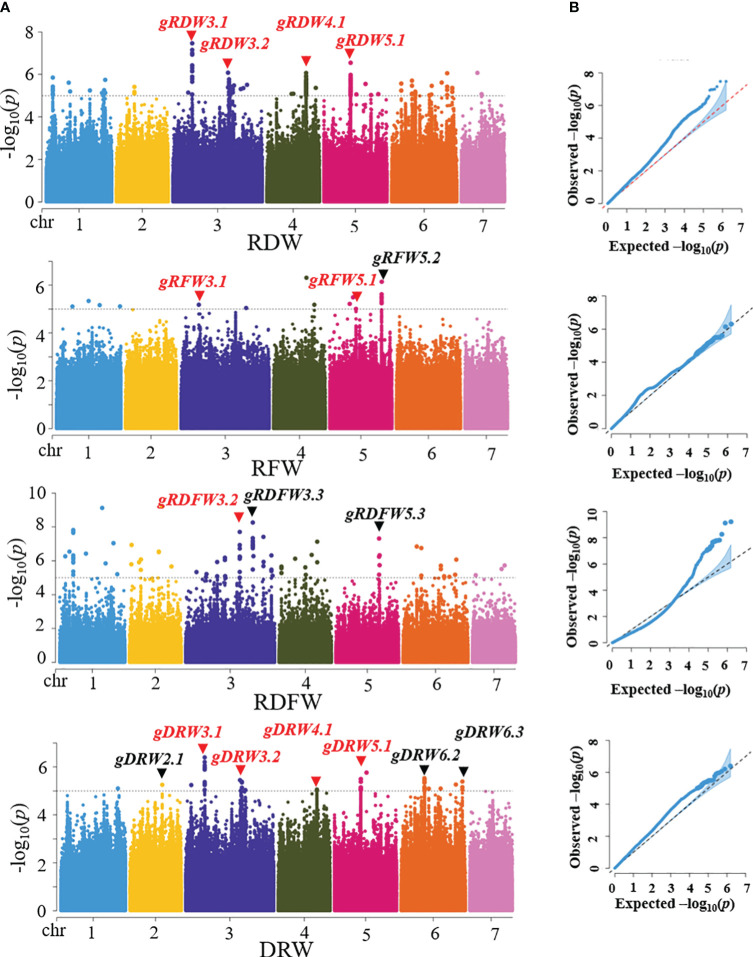
Genome-wide association analysis (GWAS) for 96 core germplasm. **(A)** Manhattan plots of RDW, RFW, RDFW, and DRW based on BLUP. Dashed line represents the significance threshold (−log_10_ *P* = 5.00). The loci marked in red were repeatedly detected for at least two root traits. **(B)** QQ plots of RDW, RFW, RDFW, and DRW based on BLUP.

The 96 core germplasm used in our study has been sequenced using Illumina platform (Qi et al., 2013) and 1,547,181 high-quality SNPs which were identified based on the ChineseLong V2.0 reference genome (http://cucurbitgenomics.org/). Based on the SNPs and phenotypic data, we used Fast-LMM (factored spectrally transformed linear mixed model) software to perform the GWAS analysis for RFW, RDW, RDFW and DRW traits to dissect the genetic basis of root-weight related traits. A total of 9 distinct loci were detected through three root traits: RFW, RDW, and RDFW (4 in RFW, 7 in RDW, and 2 in RDFW) in three experiments (2017S, 2017A, and 2020S) (**Supplementary Figures S2–4**; **Supplementary Table S5**). Then, GWAS analysis was performed based on the SNPs and BLUP data (Runcie and Crawford, 2019). A total of 10 distinct loci were detected for the four traits (4 in RDW, 3 in RFW, 3 in RDFW, and 7 in DRW). More loci information was shown in **Table 2**. Among them, 4 loci were repeatedly identified on chromosome 3, 4, and 5 for at least two traits (*gRDW3.1*, *gRDW3.2*, *gRDW4.1*, and *gRDW5.1* for RDW, *gRFW3.1*, and *gRFW5.1* for RFW, *gRDFW3.2* for RDFW, *gDRW3.1*, *gDRW3.2*, *gDRW4.1*, and *gDRW5.1* for DRW.) (**Figure 3**). Only these four stable and reliable loci were used for further analysis of candidate genes. Overall, the loci obtained from the BLUP data were highly similar as those obtained from the phenotypic data of the three seasons. (**Table 2**; **Supplementary Table S5**), indicating that these loci were repeatable and less influenced by the environment.

**Table 2:** QTLs significantly associated with root traits based on BLUP.

Traits	Loci	Peak SNP	Chromosome	Position (bp)(Chinese Long V2)	–log_10_(*P*)
RDW	***gRDW3.1***	**SNP1139794**	**3**	**8,569,196**	**7.465**
	***gRDW3.2***	**SNP1441989**	**3**	**24,511,391**	**6.079**
	***gRDW4.1***	**SNP2031473**	**4**	**17,438,778**	**6.067**
	***gRDW5.1***	**SNP2346947**	**5**	**11,837,699**	**6.546**
RFW	***gRFW3.1***	**SNP1132006**	**3**	**8,024,627**	**5.183**
	***gRFW5.1***	**SNP2346816**	**5**	**11,832,145**	**5.008**
	*gRFW5.2*	SNP2539259	5	23,377,178	6.144
RDFW	***gRDFW3.2***	**SNP1434634**	**3**	**24,163,761**	**7.701**
	*gRDFW3.3*	SNP1534956	3	29980780	8.262
	*gRDFW5.3*	SNP2471314	5	19,295,896	7.311
DRW	*gDRW2.1*	SNP858559	2	14,622,245	5.257
	***gDRW3.1***	**SNP1139794**	**3**	**8,569,196**	**6.400**
	***gDRW3.2***	**SNP1441989**	**3**	**24,511,391**	**5.451**
	***gDRW4.1***	**SNP2031473**	**4**	**17,438,778**	**5.057**
	***gDRW5.1***	**SNP2347582**	**5**	**11,858,580**	**5.496**
	*gDRW6.1*	SNP2839216	6	10,570,450	5.531
	*gDRW6.2*	SNP3143631	6	27,676,677	5.394

Bold indicates loci that have been repeatedly detected in at least two root traits.

In our study, we focused on stable and reliable loci that were identified through GWAS for additive effect genetic analysis. Interestingly, we observed that as the number of alters of heavy root increased, there was a corresponding increase in root weight. Based on these findings, we propose that the different loci identified in our study have additive effects on root size (**Supplementary Figure S5**).

### 3.3 Analysis of candidate genes associated with root weight

**Figure 4:**
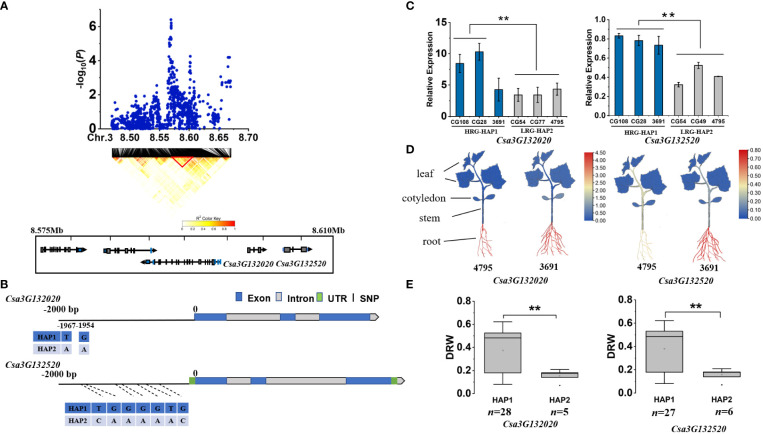
Identifying and assessing candidate genes at the *gDRW3.1* locus. **(A)** Local Manhattan plot and LD heatmap surrounding the peak (top) and genes(bottom) in LD block represented by the red tri-angle (bottom). **(B)** Gene structure of candidate gene *Csa3G132020* and *Csa3G132520*. **(C)** Relative expression levels in root of *Csa3G132020* and *Csa3G132520* in the HAP1 and HAP2 accessions through qRT-PCR. **(D)** Spatiotemporal expression of *Csa3G132020* and *Csa3G132520* in the light-root (‘4795’) and heavy-root (‘3691’) lines through qRT-PCR. **(E)** Box plots for LRG and HRG based on the haplotypes. *n* indicates the number of accessions with the same genotype. **P < 0.01 using two-sided student’s t-tests.

Since all the repeated loci were identified by GWAS using DRW, we used it for candidate gene analysis. We first determined the LD blocks harboring the peak SNP using Plink software (Purcell et al., 2007), and then identified candidate genes positioned within the LD block. Next, we used qPCR to analyze the relative expression levels (RELs) of candidate genes in different haplotype accessions and multiple tissues (root, stem, true leaf, cotyledon) of light-root ‘4795’ and heavy-root ‘3691’ accessions. Finally, we analyzed all the reference allele and alternative allele of 21 LRGS and 16 HRGS identified by aforementioned clustering analysis, and obtained different gene haplotypes based on the mutations compared with reference gemoe. The phenotypes and gene expression levels of accessions carrying different haplotypes were then statistically analyzed. For *gDRW3.1*, we identified 5 genes (*Csa3G131990*, *Csa3G132000*, *Csa3G132010*, *Csa3G132020*, *Csa3G132520*) positioned within the LD block (8.575-8.610 Mb) (**Figure 4A**). Both Csa3G132020 and Csa3G132520 had sequence variation in the promoter region (**Figure 4B**). The qRT-PCR analysis showed that the RELs of *Csa3G132020* and *Csa3G132520* both encoding F-box protein PP2-B1differed between the HAP1 and HAP2 accessions (**Figure 4C**). Notably, these two genes expressed higher in roots than in cotyledons and leaves (*P*<0.01) (**Figure 4D**). Through the haplotype analysis, for each of the two genes, we could identify two haplotypes exhibiting significantly different DRW (**Figure 4E**; **Supplementary Table S6**). As the haplotypes of two genes located in the putative promoter region (< 2.0 Kb from the start codon), we then investigated the potential effect of haplotypes on the cis-binding element using PlantCARE (http://bioinformatics.psb.ugent.be/webtools/plantcare/html/). For haplotypes of *Csa3G132020*, one of the SNPs at nucleotide position -1967 was located within the ERE element which is a typical ethylene-responsive element (**Supplementary Figure S6A**) (Shinshi, 1995; Adie et al., 2007; Liu et al., 2013). For gene *Csa3G132520*, SNP from the haplotype at nucleotide 682 was located in the CAAT-box (**Supplementary Figure S6B**). Previous studies have demonstrated that the 70-bp domain around the CAAT-box is necessary for gene expression in the dermatogen and meristematic cells of the root cortex Expression in different populations of cells of the root meristem is controlled by different domains of the rolB promoter (Capone et al., 1994).

**Figure 5:**
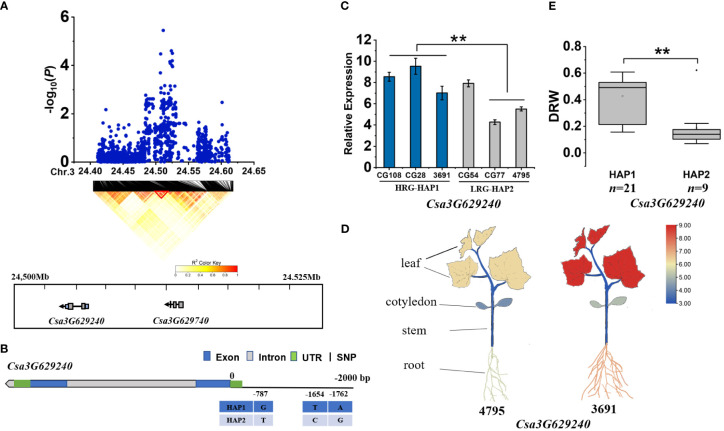
Identifying and assessing a candidate gene at the *gDRW3.2* locus. **(A)** Local Manhattan plot and LD heatmap surrounding the peak (top) and genes in LD block represented by the red tri-angle (bottom). **(B)** Gene structure of *Csa3G629240*. **(C)** Expression levels in root of *Csa3G629240* in the HAP1 and HAP2 accessions through qRT-PCR. **(D)** Spatiotemporal expression of *Csa3G629240* and *Csa3G132520* in the light-root (‘4795’) and heavy-root (‘3691’) lines through qRT-PCR. **(E)** Box plots for LRG and HRG based on the haplotypes. *n* indicates the number of accessions with the same genotype. **P < 0.01 using two-sided student’s t-tests.

For *gDRW3.2*, we identified two genes (*Csa3G629240* and *Csa3G629740*) within the LD block at 24.500-24.525 Mb on Chromosome 3 (**Figure 5A**). *Csa3G629740* was almost unexpressed in all haplotype accessions. However, the *Csa3G629240* encoding a B-cell receptor-associated protein. Three base variations exist in the promoter region of Csa3G629240 (**Figure 5B**). The expression of *Csa3G629240* in HAP1 accessions were significantly higher than that in HAP2 accessions (**Figure 5C**), and its expression in roots and leaves was higher than that in cotyledons and stem (*P*<0.01) (**Figure 5D**). The DRW of accessions with HAP1 was significantly higher than that of accessions with HAP2 (**Figure 5E**; **Supplementary Table S6**). The SNPs of haplotype at nucleotide position -1654 and -1762 on the promoter of the gene *Csa3G629240* were found to be located on the motif sequence of CAAT-box (**Supplementary Figure S7**). Thus, the above evidences all supported that *Csa3G629240* was the candidate gene for *gDRW3.2* locus.

**Figure 6:**
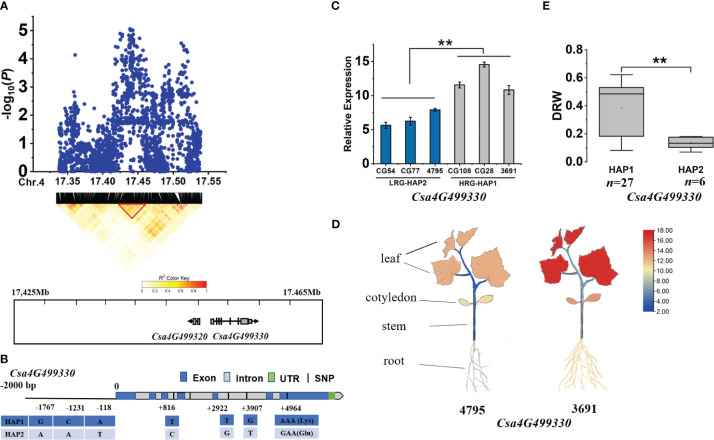
Identifying and assessing candidate genes at the *gDRW4.1* locus. **(A)** Local Manhattan plot and LD heatmap surrounding the peak (top) and Genes in LD block represented by the red tri-angle (bottom). **(B)** Gene structure of *Csa4G499330*. **(C)** Relative expression levels in root of *Csa4G499330* in the HAP1 and HAP2 accessions through qRT-PCR. **(D)** Spatiotemporal expression of *Csa4G499330* and *Csa4G499330* in the light-root (‘4795’) and heavy-root (‘3691’) lines through qRT-PCR. **(E)** Box plots for LRG and HRG based on the haplotypes. *n* indicates the number of accessions with the same genotype. **P < 0.01 using two-sided student’s t-tests.

For *gDRW4.1* locus, two candidate genes (*Csa4G499320* and *Csa4G499330*) were identified within the LD block (17.425-17.465 Mb) on Chromosome 4 (**Figure 6A**). Among them, the expression of *Csa4G499320*, encoding an unknown protein, was different at the cotyledon flattening stage. However, gene *Csa4G499330* showed significantly different expression levels between the HAP1 and HAP2 accessions (**Figure 6C**), and its expression in roots was lower than that in the cotyledons and leaves (*P*<0.01), but higher than in the stem (*P*<0.01) (**Figure 6D**). *Csa4G499330* encodes a GTP binding protein with its homolog *AT1G08410* (*Drought Inhibited Growth of Lateral roots 6*, *DIG6*) in *Arabidopsis thaliana* regulating multiple auxin-mediated developmental processes and promote lateral root development (Zhao et al., 2015). Gene-based association analysis revealed that HAP1 was mainly found in accessions with a higher DRW, while HAP2 mainly occurs in accessions with a lower DRW (**Figure 6E**; **Supplementary Table S6**). The SNP at nucleotide position +4964 on the exon caused an amino acid change, from Lys to Glu (**Figure 6B**). Therefore, through expression analysis and gene annotation, *Csa4G499330* may be promising candidate gene of *gDRW4.1*.

**Figure 7:**
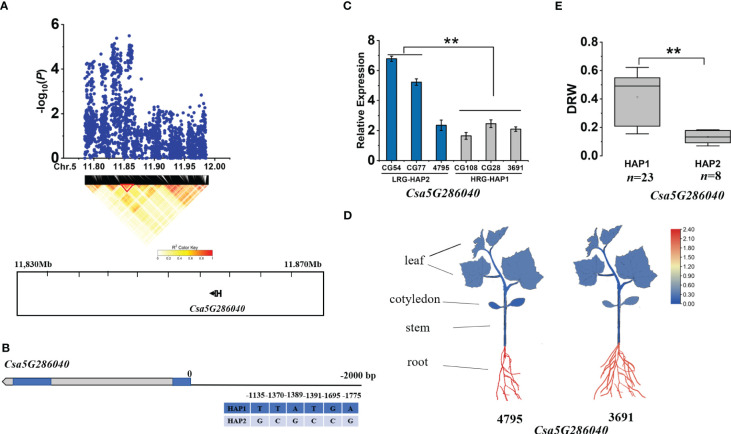
Identifying and assessing candidate genes within the *gDRW5.1* locus. **(A)** Local Manhattan plot and LD heatmap surrounding the peak (top) and Genes in LD block represented by the red tri-angle (bottom). **(B)** Gene structure of *Csa5G286040*. **(C)** Relative expression levels in root of *Csa5G286040* in HAP1 and HAP2 accessions through qRT-PCR. **(D)** Spatiotemporal expression of *Csa5G286040* and *Csa5G286040* in the light-root (‘4795’) and heavy-root (‘3691’) lines through qRT-PCR. **(E)** Box plots for LRG and HRG based on the haplotypes. *n* indicates the number of accessions with the same genotype. **P < 0.01 using two-sided student’s t-tests.

Within the *gDRW5.1*, according to the LD block, we identified one gene (*Csa5G286040*) at 11.830-11.870 Mb on Chromosome 5 (**Figure 7A**). Multiple base variations exist in the promoter region of Csa5G286040 (**Figure 7B**). The expression of *Csa5G286040* in HAP2 accessions were significantly higher than that in HAP1 accessions (**Figure 7C**). It is noteworthy that the expression level of *Csa5G286040* was higher in roots than in other tissues (**Figure 7D**). *Csa5G286040* encodes a proteinase inhibitor. The DRW of accessions with HAP1 was significantly higher than that of accessions with HAP2 (**Figure 7E**; **Supplementary Table S6**). The -1370 SNP of the promoter region is located on the G-box, and the -1389 SNP and -1391 SNP were both located within the CAAT-box (**Supplementary Figure S8**). In tomato, G-box elements are targeted by MYC2, a core TF of the jasmonic acid (JA) signaling pathway that regulates root growth (Wang et al., 2020).

## 4 Discussion

### 4.1 Genetic and phenotypic evaluation of cucumber core germplasm revealed East Asian ecotype as the elite resources for further improvement of root-weight

The study of genetic diversity is an important step in the exploration, conservation and utilization of cucumber germplasm resources. Cucumber is a shallow root vegetable crop and usually requires grafting rootstocks to improve its tolerance (Huang et al., 2010; Liu et al., 2016). At present, there are few reports about germplasm resources screening for cucumber root traits. Walters and Wehner (1994) evaluated the root length of 857 cultivated cucumber varieties. In our study, four traits related to root weight were investigated, among which RDW had the strongest correlation with DRW. So RDW is more accurate and reliable for the subjective evaluation of root weight. However, RDFW was not strongly correlated with RDW and DRW, which might be due to the difference in water content between different roots, which was also reported in other studies (Haq et al., 2008). Using the RDW index, we discovered heavy-root germplasm (16 accessions) and light-root germplasm (21 accessions), and found 56.25 percentage of heavy-root germplasm were collected from East Asian. The root weight of East Asian cucumber genotypes was also found to be significantly higher than that of other three cucumber ecotypes, thus the East Asian cucumber could be considered for use as elite resources in further breeding programs.

### 4.2 GWAS analysis of root weight

GWAS analysis of root traits have mainly focused on field crops, and there have been relatively few studies on vegetable crops like cucumber. Wang et al. (2018) analyzed SNPs from 1234 cucumber germplasm. The genetic diversity and population structure within the collection, the phylogenetic relationships, and linkage disequilibrium were characterized. Genomic regions significantly associated with 13 important horticultural traits were identified by GWAS, but no SNPs linked to root size were identified. This may be attributed to the low level of variation among root traits within the population, which would be unsuitable for a GWAS. The materials used in this study were selected from 3342 accessions collected worldwide and had a rich rate of genetic variation (Qi et al., 2013). Ten significant SNPs were detected by GWAS using four root weight-related traits.

### 4.3 Candidate genes for root weight

Root development is regulated by many genes. In this study, candidate genes were predicted for four stable loci related to root weight obtained by GWAS analysis. The excavation of root related genes is of great significance for the analysis of root development mechanism and breeding of new strong root varieties.

*Csa3G132020* and *Csa3G132520* both encode the F-box protein PP2-B1. Yan et al. (2011) found in rice that overexpression of the F-box protein gene can promote root growth. Cheah et al. (2021) found six upregulated genes in an RNA-seq study of zinc stress in rice, and speculated that the F-box protein PP2-B1 (*Os04g0280500*) might be involved in the ubiquitination and proteasome degradation of target proteins in the auxin signaling pathway that regulates root development. *Csa3G132020* and *Csa3G132520* encode homologous proteins, and their expression in the heavy-root genotype was higher than that with light-roots. Moreover, *Csa3G132020* and *Csa3G132520* were highly expressed in roots compared to other tissues. Therefore, it is speculated that these two genes may play the same role in controlling root development.

*Csa3G629240* encodes a B-cell receptor-associated protein. The function of B-cell receptors has been rarely reported in plants, however in animals, a B-cell receptor is a molecule located on the surface of B-cells responsible for the specific recognition and binding of antigens (Casola et al., 2004). In this study, the cucumber homologue *Csa3G629240* was highly expressed in roots and leaves of the heavy-root material, and may positively regulate root development.

*Csa4G499330* encodes a GTP binding protein, and its *Arabidopsis thaliana* homologue *AT1G08410* (*AtLSG1-2*) encodes a large 60S subunit nuclear output GTPase 1, which is involved in ribosomal biogenesis, and which affects development processes regulated by various auxins. The expression level of the *AtLSG1-2* was found to be highly expressed in dry seeds of *Arabidopsis thaliana*, and it exhibited a significantly higher expression in roots compared to other tissues in 1-day-old seedling (https://www.arabidopsis.org/). Zhao et al. (2015) isolated an Arabidopsis mutant *dig6*, defective in the *ATLSG1-2* gene which showed a significant reduction in the number of lateral roots. Further studies showed that *ATLSG1-2* was highly expressed in regions where the auxin accumulated, and that ribosomal biogenesis was impaired in the mutant. *ATLSG1-2* deficiency resulted in altered auxin distribution, response, and transport in plants. Thus, *ATLSG1-2* plays an indispensable role in ribosome biogenesis, with ensuing effects on auxin homeostasis and lateral root development. In this study, the expression of *Csa4G499330* in the heavy-root genotypes was higher than that in the light-root genotypes. In addition, spatiotemporal expression of *Csa4G499330* in the heavy-root genotypes was higher than that in the light-root genotypes. The expression of *Csa4G499330* in leaves was significantly higher than that in roots, which may be because it has other function in the leaves. Therefore, it is speculated that *Csa4G499330* may positively regulate plant development including root system through influencing auxin pathway.

*Csa5G286040* encodes a proteinase inhibitor. Studies have shown that in tomato, root protease inhibitors can be induced by auxin, and that auxin induction only exists in roots and hypocotyls, but not in cotyledons and hypocotyls (Taylor et al., 1993). Thus, induction may be related to the initiation of lateral and adventitious roots. Therefore, *Csa5G286040* may be induced by auxin to regulate root development. The *Csa5G286040* homologue in *Arabidopsis thaliana* is a member of the PR-6 proteinase inhibitor family, encoding a PR (disease-related) peptide. Proteinase inhibitors are activated when plants are attacked by insects, fungi, or bacteria, along with reactive oxygen species (ROS) and hormones, e.g., ethylene (ET), jasmonic acid (JA) and salicylic acid (SA). These emergency responses are usually contradictory to the normal growth and development of plants (Sels et al., 2008). In this study, in the absence of biological stress, *Csa5G286040* expression was limited to roots, and its expression in the light-root material was significantly higher than that of heavy-root material. Therefore, it can be speculated that the high expression of *Csa5G286040* in the root system disturbed the hormone balance and thus hindered root development.

## 5 Conclusions

In summary, we evaluated the phenotypic variations for the root-weight related traits of a well-known core germplasm and identified a total of 16 accessions with heavy-roots and 21 with light-roots based on a comprehensive index for the assessment of root systems which we developed, i.e., the DRW. The East Asian ecotype accessions exhibited heavier root compared with other ecotypes. Ten genomic sites related to root weight were discovered by GWAS. Further analysis indicated that *Csa3G132020*, *Csa3G132520*, *Csa3G629240*, *Csa4G499330*, and *Csa5G286040* were candidate genes that might be involved in root development. This work has identified novel cucumber cultivars that can serve as useful germplasm for breeding varieties with strong roots, furthermore, it points to candidate genes and the molecular mechanisms by which they may influence cucumber root development.

## Data availability statement

This study used the illumina data were obtained from publicly available sequence data NCBI Short Read Archive (SRA) under accession SRA056480 (http://www.ncbi.nlm.nih.gov/sra?term=SRA056480).

## Author contributions

ZD: Data curation, Software, Writing – original draft, Writing – review & editing. SD: Formal Analysis, Writing – review & editing. HC: Investigation, Methodology, Writing – review & editing. DB: Supervision, Writing – review & editing. JG: Methodology, Writing – review & editing. XL: Investigation, Methodology, Writing – review & editing. XG: Software, Writing – review & editing. HM: Data curation, Writing – review & editing. SZ: Funding acquisition, Methodology, Resources, Writing – review & editing.
