# Supplementary material for: Genome-wide association analysis reveal candidate genes and haplotypes related to root weight in cucumber (Cucumis sativus L.)
Source: Front Plant Sci. 2024 Jul 17;15:1417314. doi: 10.3389/fpls.2024.1417314 (PMC11288866; doi:10.3389/fpls.2024.1417314)
Supplement: Supplementary file 1 [file DataSheet_1.docx]

***Supplementary Material***

**Genome-wide association analysis reveal candidate genes and haplotypes related to root weight in cucumber (*Cucumis sativus* L.)**

**Table S1** Core germplasm ID and origin

| ID in this study | Name | Origin | Group of accession |  |
| --- | --- | --- | --- | --- |
| CG1 | A-C-3 | Spain, Teruel | Eurasian |  |
| CG2 | USM 307 | India, Rajasthan | Indian (Indian_2) | |
| CG3 | Tsuda | Japan | East Asian | |
| CG5 | M 5 | Netherlands | Eurasian |  |
| CG6 | NA | NA | NA |  |
| CG7 | 1972 B-2 | United States of America | Eurasian |  |
| CG8 | National Pickle | United States of America | Eurasian |  |
| CG9 | Spartan Garden MSU-C7-63 | United States of America | Eurasian |  |
| CG10 | Rasht | Iran | Eurasian |  |
| CG11 | WJR2930 | Iraq | Eurasian |  |
| CG12 | TGR 580 | Zimbabwe | Indian (Indian_2) | |
| CG13 | N2/81 | Dem. Rep. of the Congo | Eurasian |  |
| CG14 | Inyangu | Zambia, Central | Indian (Indian_2) | |
| CG15 | ZM 1065 | Zambia, Central | Indian (Indian_2) | |
| CG16 | Chibimbi | Zambia, Copperbelt | Indian (Indian_2) | |
| CG17 | USM 411 | India, Rajasthan | Indian (Indian_2) | |
| CG19 | Small Green | India | Indian (Indian_2) | |
| CG20 | KSM 588 | India, Madhya Pradesh | Indian (Indian_2) | |
| CG21 | Poona Kheera | India, Madhya Pradesh | Indian (Indian_2) | |
| CG23 | USM 321 | India, Rajasthan | Indian (Indian_2) | |
| CG25 | Xin Tai Mi Ci | NA | East Asian | |
| CG26 | 228 | NA | East Asian | |
| CG27 | Cus124/71 | China | East Asian | |
| CG28 | Kaga Fushinari | Japan | East Asian | |
| CG29 | Sagami Hanpaku Fushinari Kyuri | Japan | East Asian | |
| CG30 | NA | NA | NA |  |
| CG31 | Cgn19828 | Japan | East Asian | |
| CG32 | Hok | Netherlands | Eurasian |  |
| CG33 | CG5071 | Eurasian | Europe |  |
| CG35 | 752 | Netherlands | Eurasian |  |
| CG36 | Gy 3 (S4) | Poland | Eurasian |  |
| CG37 | Muromskij | Russian Federation | Eurasian |  |
| CG39 | NA | NA | Europe |  |
| CG40 | G8 | NA | Eurasian |  |
| CG42 | ncg042,Gl-2(NCG042) | NA | NA |  |
| CG43 | SC 53-B (6) | United States of America | Eurasian |  |
| CG44 | 2163 | United States of America | Eurasian |  |
| CG45 | SC 50 | United States of America | Eurasian |  |
| CG47 | GY14 | NA | Eurasian |  |
| CG49 | JL-14 Dhillon | India | Indian (Indian_2) | |
| CG50 | Ketimun Tjarang/Putih | Indonesia | Indian (Indian_2) | |
| CG51 | Yellow 1 | Sri Lanka | Indian (Indian_2) | |
| CG52 | NA | NA | NA |  |
| CG53 | 2003502 | NA | ? |  |
| CG54 | NA | NA | NA |  |
| CG55 | Chuan Bai | China, Sichuan | East Asian | |
| CG56 | NA | NA | NA |  |
| CG58 | NA | NA | East Asian | |
| CG59 | Ji Han | China, Jinlin | East Asian | |
| CG60 | NA | NA | NA |  |
| CG61 | NA | NA | NA |  |
| CG62 | Bai Ye San 04870 | China | East Asian | |
| CG63 | Marketmore 76 | United States of America, New York | Eurasian |  |
| CG64 | NA | NA | NA |  |
| CG66 | Ban Na Huang Gua 13 | China, Xishuangbanna | Xishuangbanna | |
| CG69 | NA | NA | NA |  |
| CG70 | Bai Chang Yuan Huang Gua | China, Xishuangbanna | Xishuangbanna | |
| CG71 | Yuan Bai Huang Gua | China, Xishuangbanna | Xishuangbanna | |
| CG72 | Bai Pi Yuan Huang Gua | China, Xishuangbanna | Xishuangbanna | |
| CG77 | Ban Na Huang Gua 51 | China, Xishuangbanna | Xishuangbanna | |
| CG78 | Ban Na Huang Gua 53 | China, Xishuangbanna | Xishuangbanna | |
| CG84 | Yuan Zong Huang Di Huang Gua | China, Xishuangbanna | Xishuangbanna | |
| CG85 | Di Huang Gua | China, Xishuangbanna | Xishuangbanna | |
| CG86 | LJ 90430 | India | Indian (Indian_1) | |
| CG87 | 13598 | India, Uttar Pradesh | Indian (Indian_2) | |
| CG88 | VIR 3147 | India | Indian (Indian_1) | |
| CG89 | CM8537-1-2-1-1-0-1-1-1 | China, Jilin | East Asian | |
| CG90 | Qing Pi Ba Cha | China, Jilin | East Asian | |
| CG91 | Da Qing7314-2-6-1-1 | China, Jilin | East Asian | |
| CG92 | Khira | Pakistan, Punjab | Indian (Indian_2) | |
| CG93 | NA | NA | NA | |
| CG94 | Liao Yang Ye San | China, Liaoning | East Asian | |
| CG95 | He Cha Huang Gua | China, Hebei | East Asian | |
| CG96 | Qian Qi Li Huang Gua | China, Hebei | East Asian | |
| CG97 | Da Ci Huang Gua | China, Sichuan | East Asian | |
| CG98 | Fu Song Ye San | China, Jilin | East Asian | |
| CG99 | Ye San Bai | China, Hebei | East Asian | |
| CG100 | Jia Huang Gua | China, Shandong | East Asian | |
| CG101 | Jin Yan Er Hao | China, Shangdong | East Asian | |
| CG102 | Qing Dao Qiu Ye Er San | China, Shandong | East Asian | |
| CG103 | Honshu Aibai | Japan, | East Asian | |
| CG104 | Sekino No. 2 (Ochiai No. | Japan, Osaka | East Asian | |
| CG105 | Qiu Huang Gua | China, Guangdong | East Asian | |
| CG106 | Leng Lu Huang Gua | China, Hunan | East Asian | |
| CG107 | 71 Hao Huang Gua | China, Chongqing | East Asian | |
| CG108 | Huang Gua | China, Shandong | East Asian | |
| CG109 | Nemet Kigyo | Hungary | Eurasian |  |
| CG110 | Kecskemeti Hamvas | Hungary | Eurasian |  |
| CG112 | Ames 1208 | Turkey, Kayseri | Eurasian |  |
| CG113 | 8181 | Turkey, Mardin | Eurasian |  |
| CG114 | Puerta-Rico #6 | Puerto Rico | Eurasian |  |
| CG117 | NA | NA | NA |  |
| CG118 | NA | NA | NA |  |
| CG120 | NA | NA | NA |  |
| CG201 | NA | NA | NA |  |

**Table S2** Phenotypic data for core germplasm

|  | 2017_Spring | | |  | 2017_Autumn | | |  | 2020_Spring | | |  | BLUP | | | |
| --- | --- | --- | --- | --- | --- | --- | --- | --- | --- | --- | --- | --- | --- | --- | --- | --- |
|  | RFW(g) | RDW(g) | RDFW |  | RFW(g) | RDW(g) | RDFW |  | RFW(g) | RDW(g) | RDFW |  | RFW(g) | RDW(g) | RDFW | DRW |
| CG1 | 0.8200 | 0.0867 | 0.105731707 |  | NA | 0.0506 | NA |  | 0.4000 | 0.0603 | 0.150666667 |  | 0.534834 | 0.065139 | 0.125564 | 0.400941 |
| CG2 | 0.4767 | 0.0767 | 0.160897839 |  | 0.6400 | 0.0534 | 0.08337963 |  | 0.2775 | 0.0624 | 0.22490991 |  | 0.513186 | 0.063544 | 0.131741 | 0.397738 |
| CG3 | 0.6000 | 0.0933 | 0.1555 |  | 0.3600 | 0.0652 | 0.181229424 |  | 0.3295 | 0.0593 | 0.179893778 |  | 0.505284 | 0.071424 | 0.1351 | 0.448096 |
| CG5 | 0.2900 | 0.0533 | 0.183793103 |  | 0.3800 | 0.0423 | 0.111413255 |  | 0.1130 | 0.0345 | 0.305530973 |  | 0.467058 | 0.044167 | 0.141056 | 0.285037 |
| CG6 | 0.6500 | 0.0900 | 0.138461538 |  | 0.4200 | 0.0575 | 0.136997354 |  | 0.4473 | 0.0629 | 0.140555142 |  | 0.522479 | 0.069122 | 0.127975 | 0.420368 |
| CG7 | NA | NA | NA |  | 0.2500 | 0.0168 | 0.067125926 |  | 0.3050 | 0.0296 | 0.097131148 |  | 0.496462 | 0.031842 | 0.118542 | 0.141523 |
| CG8 | 0.7200 | 0.0367 | 0.050972222 |  | 0.1733 | 0.0281 | 0.161868735 |  | 0.3595 | 0.0328 | 0.091318962 |  | 0.502514 | 0.034036 | 0.120055 | 0.167743 |
| CG9 | 0.5033 | 0.0733 | 0.145638784 |  | 0.3367 | 0.0567 | 0.168355168 |  | 0.2663 | 0.0463 | 0.173904881 |  | 0.49146 | 0.058515 | 0.133066 | 0.352071 |
| CG10 | 0.8233 | 0.0667 | 0.081015426 |  | 0.5250 | 0.0453 | 0.086229277 |  | 0.1797 | 0.0472 | 0.262847866 |  | 0.523282 | 0.053195 | 0.128972 | 0.339292 |
| CG11 | 0.3890 | 0.0700 | 0.179948586 |  | NA | 0.0460 | NA |  | 0.1500 | 0.0906 | 0.604166667 |  | 0.479242 | 0.067953 | 0.16578 | 0.549101 |
| CG12 | 0.4100 | 0.0833 | 0.203170732 |  | 0.5700 | 0.0541 | 0.094902534 |  | 0.3047 | 0.0527 | 0.172811816 |  | 0.50492 | 0.062788 | 0.131861 | 0.384803 |
| CG13 | 0.4110 | 0.0500 | 0.121654501 |  | NA | 0.0500 | NA |  | 0.2890 | 0.0433 | 0.149798155 |  | 0.492385 | 0.04825 | 0.126711 | 0.266179 |
| CG14 | 0.1300 | 0.0200 | 0.153846154 |  | NA | 0.0200 | NA |  | 0.2350 | 0.0198 | 0.084042553 |  | 0.465038 | 0.022273 | 0.124153 | 0.080966 |
| CG15 | NA | NA | NA |  | 0.2750 | 0.0303 | 0.110316498 |  | 0.3523 | 0.0268 | 0.075922422 |  | 0.502367 | 0.036662 | 0.120217 | 0.182684 |
| CG16 | 1.1067 | 0.0800 | 0.072286979 |  | NA | 0.0800 | NA |  | 0.3747 | 0.0500 | 0.133451957 |  | 0.55617 | 0.068993 | 0.121703 | 0.4274 |
| CG17 | NA | NA | NA |  | NA | NA | NA |  | 0.3570 | 0.0235 | 0.065826331 |  | 0.516432 | 0.033341 | 0.120201 | 0.180488 |
| CG19 | 0.9733 | 0.0467 | 0.047981095 |  | NA | 0.0467 | NA |  | 0.2660 | 0.0362 | 0.136027569 |  | 0.536409 | 0.043987 | 0.120047 | 0.260507 |
| CG20 | 0.4700 | 0.0867 | 0.184468085 |  | NA | 0.0605 | NA |  | NA | 0.0867 | NA |  | 0.5115 | 0.076409 | 0.129989 | 0.457436 |
| CG21 | 1.4200 | 0.0733 | 0.051619718 |  | 0.5667 | 0.0516 | 0.091010986 |  | 0.3570 | 0.0535 | 0.149929972 |  | 0.584847 | 0.059167 | 0.119233 | 0.394384 |
| CG23 | 0.5533 | 0.0567 | 0.102476053 |  | NA | 0.0471 | NA |  | 0.6820 | 0.0456 | 0.06686217 |  | 0.536083 | 0.050161 | 0.118929 | 0.288351 |
| CG25 | 0.5867 | 0.1067 | 0.181864667 |  | 0.4167 | 0.0746 | 0.179119004 |  | 0.5643 | 0.0705 | 0.125011077 |  | 0.526277 | 0.082015 | 0.132931 | 0.519142 |
| CG26 | 1.1167 | 0.0767 | 0.068684517 |  | 0.5050 | 0.0613 | 0.121309131 |  | 0.5403 | 0.0564 | 0.104303563 |  | 0.57113 | 0.064115 | 0.119356 | 0.406366 |
| CG27 | NA | NA | NA |  | NA | 0.0190 | NA |  | 0.1535 | 0.0420 | 0.273615635 |  | 0.498343 | 0.038428 | 0.137343 | 0.271208 |
| CG28 | 0.8233 | 0.1233 | 0.149763148 |  | 0.3133 | 0.0735 | 0.234611247 |  | 0.3337 | 0.0795 | 0.238336663 |  | 0.518927 | 0.089618 | 0.142612 | 0.599434 |
| CG29 | 0.6133 | 0.0567 | 0.092450677 |  | 0.4933 | 0.0404 | 0.081980014 |  | 0.2548 | 0.0389 | 0.152747792 |  | 0.510707 | 0.045999 | 0.121685 | 0.250236 |
| CG30 | 0.3600 | 0.0700 | 0.194444444 |  | 0.6567 | 0.0573 | 0.087200875 |  | 0.3130 | 0.0479 | 0.153154952 |  | 0.508318 | 0.058173 | 0.129306 | 0.351046 |
| CG31 | 1.2233 | 0.0767 | 0.062699256 |  | 0.5067 | 0.0493 | 0.097245064 |  | 0.3130 | 0.0555 | 0.177236422 |  | 0.562152 | 0.060115 | 0.122393 | 0.389151 |
| CG32 | 0.6067 | 0.0667 | 0.109939014 |  | 0.2300 | 0.0402 | 0.174766506 |  | 0.3388 | 0.0485 | 0.143284133 |  | 0.496677 | 0.052026 | 0.128823 | 0.30194 |
| CG33 | 0.7600 | 0.1033 | 0.135921053 |  | 0.2700 | 0.0647 | 0.239643347 |  | 0.5768 | 0.0653 | 0.113285652 |  | 0.529228 | 0.076251 | 0.133133 | 0.492054 |
| CG35 | 0.2800 | 0.0367 | 0.131071429 |  | 0.1600 | 0.0267 | 0.166736111 |  | 0.6170 | 0.0285 | 0.046252026 |  | 0.487737 | 0.032275 | 0.12288 | 0.155107 |
| CG36 | 1.4833 | 0.0933 | 0.06290029 |  | 0.4133 | 0.0581 | 0.140580334 |  | 0.5643 | 0.0648 | 0.114798405 |  | 0.593688 | 0.070914 | 0.121055 | 0.477366 |
| CG37 | 0.2790 | 0.0600 | 0.215053763 |  | 0.8600 | 0.0413 | 0.048072782 |  | 0.3818 | 0.0428 | 0.111984283 |  | 0.522737 | 0.048499 | 0.125079 | 0.294088 |
| CG39 | 0.9767 | 0.1067 | 0.109245418 |  | NA | 0.0676 | NA |  | NA | 0.1067 | NA |  | 0.55654 | 0.091061 | 0.123783 | 0.558293 |
| CG40 | 0.8433 | 0.0600 | 0.071149057 |  | 0.5933 | 0.0558 | 0.094125138 |  | 0.5148 | 0.0386 | 0.075036425 |  | 0.555235 | 0.051726 | 0.115534 | 0.302107 |
| CG42 | 0.4033 | 0.0367 | 0.090999256 |  | NA | 0.0250 | NA |  | NA | 0.0367 | NA |  | 0.505571 | 0.034291 | 0.122278 | 0.183443 |
| CG43 | 0.5200 | 0.0633 | 0.121730769 |  | 0.2750 | 0.0402 | 0.146188552 |  | 0.4987 | 0.0489 | 0.098061497 |  | 0.505599 | 0.051083 | 0.124432 | 0.285541 |
| CG44 | 0.8167 | 0.0467 | 0.05718134 |  | NA | 0.0321 | NA |  | 0.3063 | 0.0335 | 0.109510204 |  | 0.526911 | 0.038623 | 0.118728 | 0.214023 |
| CG45 | 0.7800 | 0.0567 | 0.072692308 |  | 0.4233 | 0.0469 | 0.110901121 |  | 0.4150 | 0.0465 | 0.111987952 |  | 0.5301 | 0.050373 | 0.119447 | 0.285229 |
| CG47 | 0.7133 | 0.0833 | 0.116781158 |  | 0.5233 | 0.0601 | 0.114826847 |  | NA | 0.0833 | NA |  | 0.538349 | 0.074183 | 0.123675 | 0.445046 |
| CG49 | 0.1467 | 0.0333 | 0.226993865 |  | 0.1250 | 0.0270 | 0.216 |  | 0.2713 | 0.0299 | 0.110350123 |  | 0.448947 | 0.031754 | 0.1377 | 0.180399 |
| CG50 | 0.5987 | 0.0567 | 0.094705195 |  | NA | 0.0394 | NA |  | 0.1717 | 0.0391 | 0.227524271 |  | 0.498129 | 0.045723 | 0.130581 | 0.277789 |
| CG51 | 0.3467 | 0.0400 | 0.115373522 |  | 0.6033 | 0.0356 | 0.059054828 |  | 0.5110 | 0.0305 | 0.059727658 |  | 0.518228 | 0.0367 | 0.115098 | 0.175974 |
| CG52 | 0.4833 | 0.0367 | 0.075936271 |  | 0.5067 | 0.0440 | 0.086876594 |  | NA | 0.0367 | NA |  | 0.518219 | 0.040205 | 0.118432 | 0.211329 |
| CG53 | 0.5067 | 0.0367 | 0.072429445 |  | 0.1500 | 0.0252 | 0.167950617 |  | 0.8568 | 0.0364 | 0.04250073 |  | 0.522186 | 0.034261 | 0.118548 | 0.183992 |
| CG54 | 0.0933 | 0.0167 | 0.178992497 |  | 0.1750 | 0.0163 | 0.093216931 |  | NA | 0.0167 | NA |  | 0.459304 | 0.019152 | 0.126769 | 0.070176 |
| CG55 | 0.3757 | 0.0800 | 0.212935853 |  | 0.3733 | 0.0579 | 0.155033684 |  | 0.6793 | 0.0573 | 0.084284137 |  | 0.515756 | 0.064367 | 0.130541 | 0.399306 |
| CG56 | 0.4300 | 0.0567 | 0.131860465 |  | 0.3250 | 0.0417 | 0.128250712 |  | 0.3818 | 0.0404 | 0.105861166 |  | 0.493756 | 0.046852 | 0.124432 | 0.249001 |
| CG58 | 0.3867 | 0.0867 | 0.22420481 |  | 0.7067 | 0.0595 | 0.084136493 |  | 0.7115 | 0.0636 | 0.089388616 |  | 0.544183 | 0.068918 | 0.126681 | 0.437622 |
| CG59 | 1.8333 | 0.1133 | 0.061801124 |  | 0.5400 | 0.0701 | 0.129845679 |  | 0.9283 | 0.0764 | 0.082253142 |  | 0.657144 | 0.084471 | 0.117912 | 0.608119 |
| CG60 | 0.4267 | 0.0700 | 0.164049684 |  | NA | 0.0514 | NA |  | 0.6057 | 0.0480 | 0.079182719 |  | 0.519517 | 0.056348 | 0.124561 | 0.3307 |
| CG61 | 0.8667 | 0.0567 | 0.065420561 |  | NA | 0.0384 | NA |  | NA | 0.0567 | NA |  | 0.546763 | 0.050888 | 0.120168 | 0.310493 |
| CG62 | 0.3567 | 0.0567 | 0.158957107 |  | 0.2533 | 0.0459 | 0.181251919 |  | 0.4438 | 0.0436 | 0.098253521 |  | 0.487492 | 0.049158 | 0.129565 | 0.279474 |
| CG63 | 1.1267 | 0.1033 | 0.091683678 |  | NA | 0.0650 | NA |  | 0.4323 | 0.0640 | 0.147975709 |  | 0.562503 | 0.075916 | 0.124288 | 0.484949 |
| CG64 | 0.3933 | 0.0267 | 0.067887109 |  | NA | 0.0267 | NA |  | 0.1198 | 0.0173 | 0.14453723 |  | 0.477124 | 0.02568 | 0.122213 | 0.103829 |
| CG66 | NA | NA | NA |  | NA | 0.0140 | NA |  | NA | NA | NA |  | 0.462163 | 0.027132 | NA | NA |
| CG69 | 0.8633 | 0.0833 | 0.096490212 |  | 0.2767 | 0.0544 | 0.196718267 |  | NA | 0.0567 | NA |  | 0.530464 | 0.064137 | 0.128369 | 0.404181 |
| CG70 | NA | NA | NA |  | NA | NA | NA |  | 0.5443 | 0.0295 | 0.054279589 |  | 0.533076 | 0.03831 | 0.119249 | 0.221863 |
| CG71 | NA | NA | NA |  | NA | NA | NA |  | 0.7275 | 0.0395 | 0.054295533 |  | NA | 0.046499 | 0.11925 | 0.285048 |
| CG72 | 0.8300 | 0.0600 | 0.072289157 |  | NA | 0.0600 | NA |  | 0.3215 | 0.0460 | 0.143079316 |  | 0.529242 | 0.055311 | 0.122437 | 0.325755 |
| CG77 | 0.3500 | 0.0333 | 0.095142857 |  | NA | 0.0333 | NA |  | 0.2307 | 0.0243 | 0.105238439 |  | 0.482643 | 0.031951 | 0.121295 | 0.139826 |
| CG78 | 1.3200 | 0.1200 | 0.090909091 |  | 0.4967 | 0.0865 | 0.174164299 |  | 0.3948 | 0.0783 | 0.198226726 |  | NA | 0.092239 | 0.131324 | 0.622305 |
| CG84 | NA | NA | NA |  | NA | NA | NA |  | 0.5788 | 0.0358 | 0.061771058 |  | 0.536143 | 0.043415 | 0.119867 | 0.256204 |
| CG85 | 0.6467 | 0.0767 | 0.118602134 |  | 0.3700 | 0.0597 | 0.161301301 |  | 0.4583 | 0.0564 | 0.122945455 |  | 0.519287 | 0.063623 | 0.127043 | 0.382212 |
| CG86 | NA | 0.0200 | NA |  | NA | 0.0124 | NA |  | 0.0575 | 0.0125 | 0.217391304 |  | 0.489809 | 0.01764 | 0.132705 | 0.125543 |
| CG87 | NA | NA | NA |  | NA | NA | NA |  | 0.4665 | 0.0279 | 0.059753483 |  | 0.526165 | 0.036939 | 0.1197 | 0.208732 |
| CG88 | 0.6100 | 0.0567 | 0.09295082 |  | NA | 0.0567 | NA |  | 0.4388 | 0.0434 | 0.098945869 |  | 0.520854 | 0.052454 | 0.120649 | 0.291922 |
| CG89 | 1.1333 | 0.0800 | 0.070590311 |  | 0.6300 | 0.0524 | 0.083104056 |  | 0.4493 | 0.0558 | 0.124095715 |  | 0.574949 | 0.062185 | 0.118187 | 0.394488 |
| CG90 | 0.3833 | 0.0867 | 0.226193582 |  | 0.5567 | 0.0574 | 0.103051048 |  | 0.4043 | 0.0559 | 0.138260153 |  | 0.509416 | 0.065871 | 0.131621 | 0.405562 |
| CG91 | 0.6533 | 0.1067 | 0.163324659 |  | 0.7233 | 0.0750 | 0.103701015 |  | 0.8505 | 0.0711 | 0.083548893 |  | 0.576047 | 0.082291 | 0.123341 | 0.530495 |
| CG92 | 0.4333 | 0.0667 | 0.153934918 |  | 0.6667 | 0.0482 | 0.072285275 |  | 0.7628 | 0.0512 | 0.067158309 |  | 0.548549 | 0.055348 | 0.119291 | 0.332571 |
| CG93 | NA | NA | NA |  | NA | NA | NA |  | 0.2893 | 0.0196 | 0.067847882 |  | 0.510409 | 0.030154 | 0.120368 | 0.157076 |
| CG94 | 0.7467 | 0.1033 | 0.138342038 |  | 0.6000 | 0.0720 | 0.119938272 |  | 0.4830 | 0.0670 | 0.138724983 |  | 0.546054 | 0.079027 | 0.126629 | 0.49462 |
| CG95 | 1.4100 | 0.1100 | 0.078014184 |  | 0.5600 | 0.0779 | 0.139189815 |  | 0.4210 | 0.0757 | 0.17982977 |  | 0.588417 | 0.085677 | 0.126631 | 0.579058 |
| CG96 | 0.6733 | 0.1000 | 0.148522204 |  | 0.3200 | 0.0684 | 0.213755787 |  | 0.7415 | 0.0739 | 0.099713419 |  | 0.538892 | 0.079049 | 0.131231 | 0.509029 |
| CG97 | 0.5867 | 0.0567 | 0.09659091 |  | 0.6467 | 0.0426 | 0.065924437 |  | 0.5498 | 0.0423 | 0.077004699 |  | 0.542539 | 0.047734 | 0.115478 | 0.265633 |
| CG98 | 0.3600 | 0.0750 | 0.208333333 |  | 0.2450 | 0.0510 | 0.20824641 |  | 0.3563 | 0.0479 | 0.134471469 |  | NA | 0.057779 | 0.137537 | 0.357415 |
| CG99 | 0.5600 | 0.0500 | 0.089285714 |  | 0.5700 | 0.0466 | 0.081769006 |  | 0.4998 | 0.0433 | 0.086543272 |  | 0.530964 | 0.047182 | 0.116758 | 0.255703 |
| CG100 | NA | NA | NA |  | NA | 0.0220 | NA |  | 0.3845 | 0.0258 | 0.066970091 |  | 0.518876 | 0.032448 | 0.120296 | 0.178859 |
| CG101 | NA | 0.0833 | NA |  | 0.2650 | 0.0571 | 0.215443746 |  | 0.5700 | 0.0586 | 0.102785088 |  | 0.519319 | 0.065566 | 0.130276 | 0.408601 |
| CG102 | 0.4967 | 0.0633 | 0.127441111 |  | 0.5300 | 0.0543 | 0.102473795 |  | 0.4527 | 0.0476 | 0.105219072 |  | 0.519614 | 0.055075 | 0.122248 | 0.312598 |
| CG103 | 0.6467 | 0.0867 | 0.134065254 |  | 0.4867 | 0.0604 | 0.124169577 |  | NA | 0.0867 | NA |  | 0.529925 | 0.076404 | 0.125704 | 0.457473 |
| CG104 | 0.5867 | 0.0733 | 0.124936083 |  | 0.6600 | 0.0581 | 0.08798541 |  | 0.7203 | 0.0561 | 0.077834336 |  | 0.556419 | 0.061977 | 0.119106 | 0.376763 |
| CG105 | 0.5633 | 0.0700 | 0.124267708 |  | 0.5667 | 0.0455 | 0.080256717 |  | 1.1200 | 0.0598 | 0.053348214 |  | 0.577775 | 0.058182 | 0.116777 | 0.368998 |
| CG106 | 0.3767 | 0.0800 | 0.212370587 |  | 1.1100 | 0.0669 | 0.060228562 |  | 1.1328 | 0.0668 | 0.058982565 |  | 0.605658 | 0.070133 | 0.121997 | 0.491309 |
| CG107 | 1.0000 | 0.0867 | 0.0867 |  | 0.6100 | 0.0562 | 0.092082574 |  | 0.8190 | 0.0624 | 0.076205739 |  | 0.591285 | 0.067526 | 0.116573 | 0.434317 |
| CG108 | 0.5400 | 0.1000 | 0.185185185 |  | 0.2900 | 0.0680 | 0.234527458 |  | 0.7510 | 0.0669 | 0.089131158 |  | 0.527284 | 0.076752 | 0.134549 | 0.499474 |
| CG109 | 0.3500 | 0.0833 | 0.238 |  | NA | 0.0565 | NA |  | 1.0307 | 0.0613 | 0.059492238 |  | 0.54795 | 0.066229 | 0.128696 | 0.437062 |
| CG110 | 0.4367 | 0.0733 | 0.167849782 |  | 0.4667 | 0.0509 | 0.10911919 |  | 0.5110 | 0.0533 | 0.104378669 |  | 0.514711 | 0.058907 | 0.125521 | 0.343869 |
| CG112 | 0.7567 | 0.0933 | 0.123298533 |  | 0.3400 | 0.0590 | 0.173480392 |  | 1.0245 | 0.0724 | 0.070668619 |  | 0.568054 | 0.073559 | 0.124536 | 0.479616 |
| CG113 | 0.8933 | 0.0533 | 0.059666405 |  | 0.2667 | 0.0375 | 0.140419948 |  | NA | 0.0533 | NA |  | 0.532096 | 0.048486 | 0.121273 | 0.286105 |
| CG114 | 0.8167 | 0.0700 | 0.085710787 |  | 0.2333 | 0.0446 | 0.191289232 |  | 0.4438 | 0.0500 | 0.112676056 |  | 0.520699 | 0.054885 | 0.12611 | 0.331618 |
| CG117 | 0.3967 | 0.0467 | 0.1177212 |  | NA | 0.0467 | NA |  | 1.1078 | 0.0484 | 0.043647032 |  | 0.558054 | 0.047771 | 0.118322 | 0.297336 |
| CG118 | 0.9933 | 0.0700 | 0.070472163 |  | 0.5850 | 0.0500 | 0.085448982 |  | 0.3070 | 0.0556 | 0.181188925 |  | 0.550251 | 0.0583 | 0.122388 | 0.365699 |
| CG120 | 0.5867 | 0.0667 | 0.113686722 |  | 0.4267 | 0.0495 | 0.116011285 |  | 0.4743 | 0.0508 | 0.107090142 |  | 0.520237 | 0.055618 | 0.122365 | 0.316836 |
| CG201 | NA | NA | NA |  | NA | NA | NA |  | 0.4000 | 0.0250 | 0.0625 |  | 0.520254 | 0.034575 | 0.119927 | 0.190223 |

Table S3 Primer information

| Primer name | Forward(5’-3’) | Reverse(5’-3’) |
| --- | --- | --- |
| *Csa3G131990* | GGTGGAAGAAGCCAAAGTT | CGTCAGAGGCATCCTTAGA |
| *Csa3G132000* | GACCCCACACGACTTCAT | ACCAGTTCCATCGCATTC |
| *Csa3G132010* | GTCGCTCTCAAGTGTGGAG | CAACCTGCTTCAATTCGC |
| *Csa3G132020* | GATCTGGACATTATTTGGGG | TTCAAGCCACCACACAAC |
| *Csa3G132520* | CAAGTTTAAGGAAGGCGATT | ACCATTTTCGTTCCATCTTC |
| *Csa3G629240* | GCTGCTACCATGTCTGTCAT | GGTAAAACCAATGAGTGAAGC |
| *Csa3G629740* | TCCCAACACAGACGGAAT | TTTTACAGTCAAGGAACCAGG |
| *Csa4G499320* | GATGCTAGGGTTGTTGATGTT | CATGAAAGTCAATAGATCCCAC |
| *Csa4G499330* | AACACAAGAGGACAATGCTTC | TTTTCCCATCCAGAGTGG |
| *Csa5G286040* | GCTGATATTTGTCCTCCTGG | GTTCCACTTCCAGCCAAG |

Table S4 PCA results

|  | RDW | RFW | RDFW | Cumulative contribution rate | Contribution |
| --- | --- | --- | --- | --- | --- |
| Prin1 | 0.599944 | 0.757131 | -0.2585 | 0.5352 | 0.5352 |
| Prin2 | 0.546757 | -0.15214 | 0.823353 | 0.9586 | 0.4234 |
| Prin3 | -0.58406 | 0.6353 | 0.505242 | 1 | 0.0414 |

Table S5. QTLs significantly associated with RFW, RDW, and RDFW

| Traits | Environment | Loci | Peak SNP | Chromosome | Position (bp) | –log_10_(*P*) |
| --- | --- | --- | --- | --- | --- | --- |
| RFW | 2017S | *gRFW3.1* | SNP1131997 | 3 | 8024380 | 5.594208905 |
|  | 2017S | *gRFW5.2* | SNP2538144 | 5 | 23314712 | 5.765195064 |
|  | 2020S | *gRFW6.1* | SNP2825417 | 6 | 9847447 | 6.071349716 |
| RDW | 2017S | *gRDW3.1* | SNP1139804 | 3 | 8569523 | 7.279027027 |
|  | 2017S | *gRDW3.2* | SNP1450125 | 3 | 24884634 | 5.70415557 |
|  | 2017S | *gRDW4.1* | SNP2031473 | 4 | 17438778 | 6.330809145 |
|  | 2017S | *gRDW5.1* | SNP2347582 | 5 | 11858580 | 5.521012888 |
|  | 2017S | *gRDW6.1* | SNP2839216 | 6 | 10570450 | 6.460505823 |
|  | 2017A | *gRDW3.1* | SNP1139815 | 3 | 8569733 | 6.64068995 |
|  | 2017A | *gRDW4.1* | SNP2031439 | 4 | 17437524 | 7.078206991 |
|  | 2017A | *gRDW5.1* | SNP2346947 | 5 | 11837699 | 6.893883877 |
|  | 2017A | *gRDW6.2* | SNP3132818 | 6 | 26967895 | 5.78817931 |
|  | 2020S | *gRDW2.1* | SNP858575 | 2 | 14622887 | 5.198653352 |
|  | 2020S | *gRDW3.1* | SNP1139794 | 3 | 8569196 | 6.26621147 |
|  | 2020S | *gRDW3.2* | SNP1451557 | 3 | 24942278 | 5.668542527 |
|  | 2020S | *gRDW4.1* | SNP2031102 | 4 | 17424710 | 5.25824147 |
|  | 2020S | *gRDW5.1* | SNP2347582 | 5 | 11858580 | 5.702758132 |
|  | 2020S | *gRDW6.1* | SNP2839291 | 6 | 10573471 | 5.681830456 |
|  | 2020S | *gRDW6.2* | SNP3143631 | 6 | 27676677 | 5.94685144 |
| RDFW | 2020S | *gRDFW3.2* | SNP1436485 | 3 | 24246382 | 12.27898888 |
|  | 2020S | *gRDFW5.3* | SNP2471938 | 5 | 19327006 | 11.44516518 |

Table S6 SNP haplotypes of the LRG and HRG

|  |  | Csa3G132020 | | Csa3G132520 | | | | | | | | Csa3G629240 | | | Csa4G499330 | | | | | | | Csa5G286040 | | | | | | Phenotype |
| --- | --- | --- | --- | --- | --- | --- | --- | --- | --- | --- | --- | --- | --- | --- | --- | --- | --- | --- | --- | --- | --- | --- | --- | --- | --- | --- | --- | --- |
|  |  | Chr3 | Chr3 | Chr3 | Chr3 | Chr3 | Chr3 | Chr3 | Chr3 | Chr3 | Chr3 | Chr3 | Chr3 | Chr3 | Chr4 | Chr4 | Chr4 | Chr4 | Chr4 | Chr4 | Chr4 | Chr5 | Chr5 | Chr5 | Chr5 | Chr5 | Chr5 |  |
|  | Position(bp) | 8599050 | 8599063 | 8603454 | 8603464 | 8603893 | 8604029 | 8604438 | 8604562 | 8604632 | 8604761 | 24507087 | 24507954 | 24508062 | 17447228 | 17447764 | 17448877 | 17449811 | 17451917 | 17452902 | 17453959 | 11858326 | 11858561 | 11858580 | 11858582 | 11858887 | 11858966 |  |
|  | Ref | T | G | T | G | G | G | G | T | G | G | G | T | A | G | C | A | T | T | G | A | T | T | A | T | G | A |  |
| LRG | CG100 | T | G | T | G | G | G | G | T | G | G | G | T | A | G | C | A | T | T | G | A | T | T | A | T | G | A | 0.178859 |
|  | CG14 | T | G | T | G | G | G | G | T | G | G | T | C | G | G | C | A | T | T | G | A | G | C | G | C | C | G | 0.080966 |
|  | CG15 | T | G | T | G | G | G | G | T | A | G | K | Y | - | G | C | A | T | T | G | A | G | C | G | C | C | G | 0.182684 |
|  | CG17 | W | R | Y | R | A | R | A | A | A | C | K | T | - | G | C | A | T | T | G | A | - | Y | - | Y | S | - | 0.180488 |
|  | CG201 | T | G | T | G | G | G | G | T | G | G | G | T | A | G | C | A | T | T | G | A | T | T | A | T | G | A | 0.190223 |
|  | CG35 | T | G | T | G | G | G | G | T | G | G | T | C | G | G | C | A | T | T | G | A | T | T | A | T | G | A | 0.155107 |
|  | CG44 | T | G | T | G | G | G | G | T | G | G | G | T | A | G | C | A | T | T | G | A | T | T | A | T | G | A | 0.214023 |
|  | CG49 | T | G | C | A | A | A | A | A | A | C | G | T | A | A | A | T | C | G | T | G | G | C | G | C | C | G | 0.180399 |
|  | CG51 | W | R | Y | R | R | R | R | W | R | S | T | C | G | A | A | T | C | G | T | G | G | C | G | C | C | G | 0.175974 |
|  | CG54 | A | A | C | A | A | A | A | A | A | C | T | C | G | A | A | T | C | G | T | G | G | C | G | C | C | G | 0.070176 |
|  | CG64 | T | G | T | G | G | G | G | T | G | G | T | C | G | A | A | T | C | G | T | G | G | C | G | C | C | G | 0.103829 |
|  | CG70 | T | G | T | G | G | G | G | T | G | G | T | C | G | G | C | A | T | T | G | A | T | T | A | T | G | A | 0.221863 |
|  | CG77 | A | A | C | A | A | R | A | A | A | C | T | C | G | A | A | T | C | G | T | G | G | C | G | C | C | G | 0.139826 |
|  | CG8 | T | G | T | G | G | G | G | T | G | G | G | T | A | G | C | A | T | T | G | A | T | T | A | T | G | A | 0.167743 |
|  | CG86 | T | G | T | G | G | G | G | T | G | G | T | C | G | A | A | T | C | G | T | G | G | C | G | C | C | G | 0.125543 |
|  | CG87 | W | R | Y | R | R | R | R | W | G | S | K | T | - | G | C | A | T | T | G | A | T | T | A | T | G | A | 0.208732 |
|  | CG93 | T | G | T | G | G | G | G | T | G | G | G | T | A | G | C | A | T | T | G | A | T | T | A | T | G | A | 0.157076 |
| HRG | CG106 | T | G | T | G | G | G | G | T | G | G | G | T | A | G | C | A | T | T | G | A | T | T | A | T | G | A | 0.491309 |
|  | CG108 | T | G | T | G | G | G | G | T | G | G | G | T | A | G | C | A | T | T | G | A | T | T | A | T | G | A | 0.499474 |
|  | CG11 | T | G | T | G | G | G | G | T | G | G | G | T | A | G | C | A | T | T | G | A | T | T | A | T | G | A | 0.549101 |
|  | CG112 | T | G | T | G | G | G | G | T | G | G | G | T | A | G | C | A | T | T | G | A | T | T | A | T | G | A | 0.479616 |
|  | CG25 | - | - | - | G | - | G | - | - | - | G | G | T | - | G | - | A | T | - | G | A | T | - | - | - | G | - | 0.519142 |
|  | CG28 | T | G | T | G | G | G | G | T | G | G | G | T | A | G | C | A | T | T | G | A | T | T | A | T | G | A | 0.599434 |
|  | CG33 | T | G | T | G | G | G | G | T | G | G | G | T | A | G | C | A | T | T | G | - | T | T | A | T | G | A | 0.492054 |
|  | CG36 | T | G | T | G | G | G | G | T | G | - | G | T | A | G | C | A | T | T | G | A | T | T | A | T | G | A | 0.477366 |
|  | CG39 | T | G | T | G | G | G | G | T | - | G | G | T | A | G | C | A | T | T | G | A | T | T | A | T | G | A | 0.558293 |
|  | CG59 | T | G | T | G | G | G | G | T | G | G | G | T | A | G | C | A | T | T | G | A | T | T | A | T | G | A | 0.608119 |
|  | CG63 | - | - | - | - | - | - | - | T | G | G | G | T | A | G | C | A | T | T | G | A | T | T | A | T | G | A | 0.484949 |
|  | CG78 | T | G | T | G | G | G | G | T | G | G | T | C | G | G | C | A | T | T | G | A | T | T | A | T | G | A | 0.622305 |
|  | CG91 | T | G | T | G | G | G | G | T | G | G | G | T | A | G | C | A | T | T | G | A | T | T | A | T | G | A | 0.530495 |
|  | CG94 | T | G | T | G | G | G | G | T | G | G | G | T | A | G | C | A | T | T | G | A | T | T | A | T | G | A | 0.49462 |
|  | CG95 | T | G | T | G | G | G | G | T | G | G | G | T | A | G | C | A | T | T | G | A | T | T | A | T | G | A | 0.579058 |
|  | CG96 | T | G | T | G | G | G | G | T | G | G | G | T | A | G | C | A | T | T | G | A | T | T | A | T | G | A | 0.509029 |


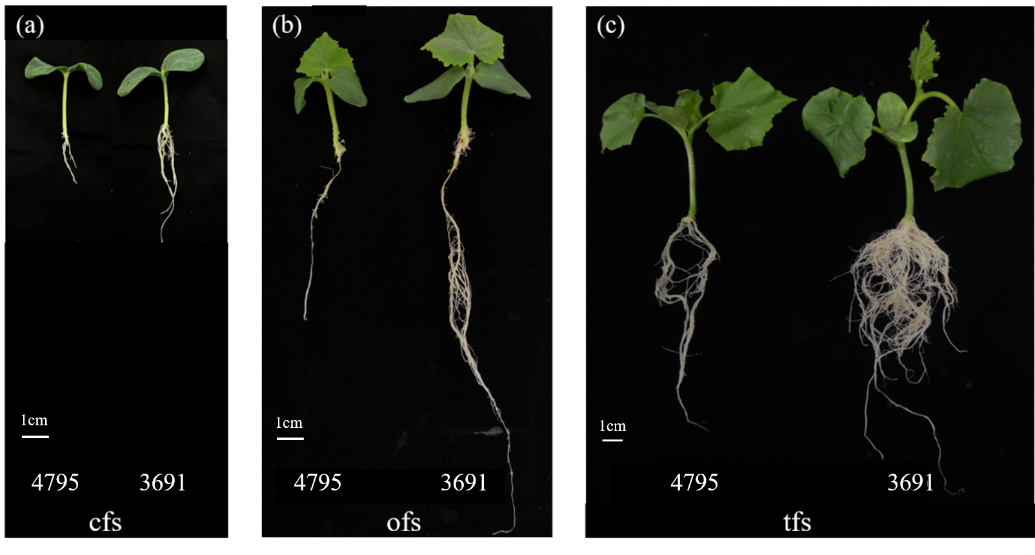


**Figure S1. Phenotypes of light-root (4795) and heavy-root (3691) line at the cotyledon flattening stage (cfs), one true leaf flattening stage (ofs), and two true leaves flattening stage (tfs).**


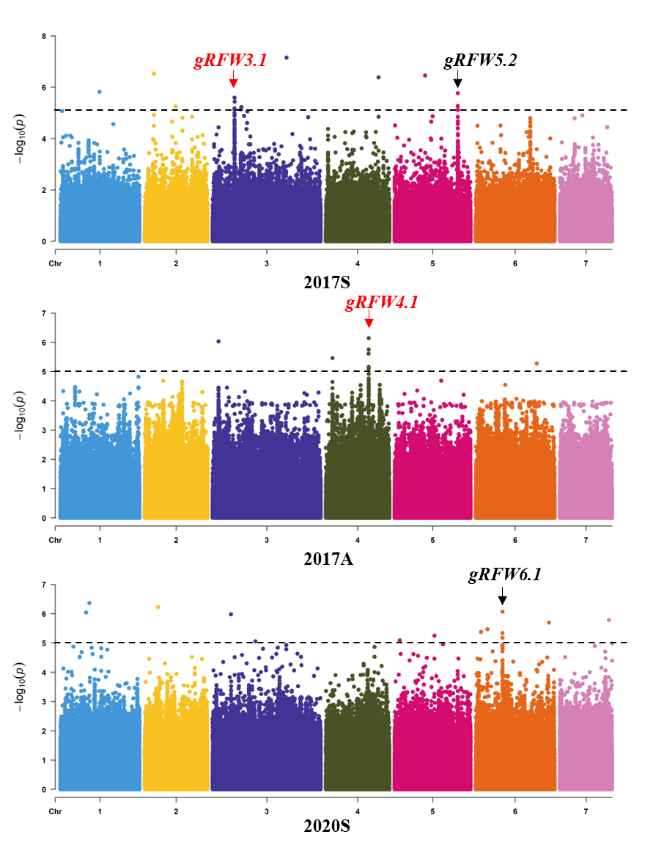


**Figure S2. Genome-wide association analysis (GWAS) of root fresh weight for 96 core germplasm.** **Dashed line represents the significance threshold (−log_10_ *P* = 5.00)**


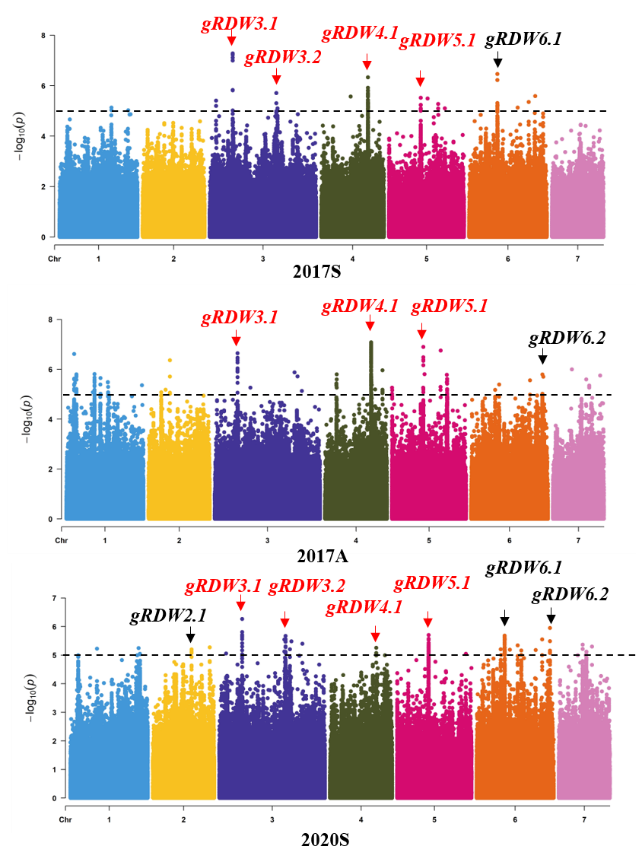


**Figure S3. Genome-wide association analysis (GWAS) of root dry weight for 96 core germplasm.** **Dashed line represents the significance threshold (−log_10_ *P* = 5.00)**


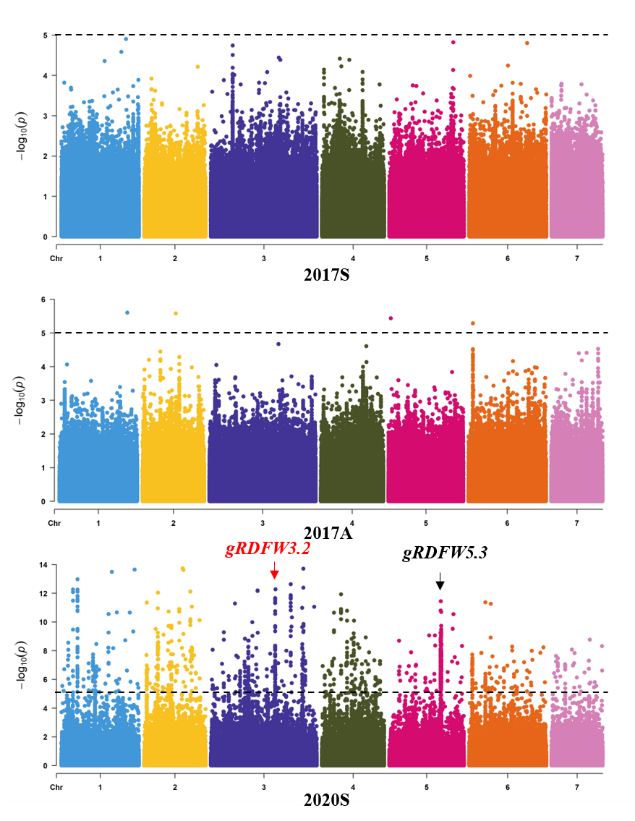


**Figure S4. Genome-wide association analysis (GWAS) of ratio of dry root weight to fresh root weight for 96 core germplasm. Dashed line represents the significance threshold (−log_10_ *P* = 5.00)**


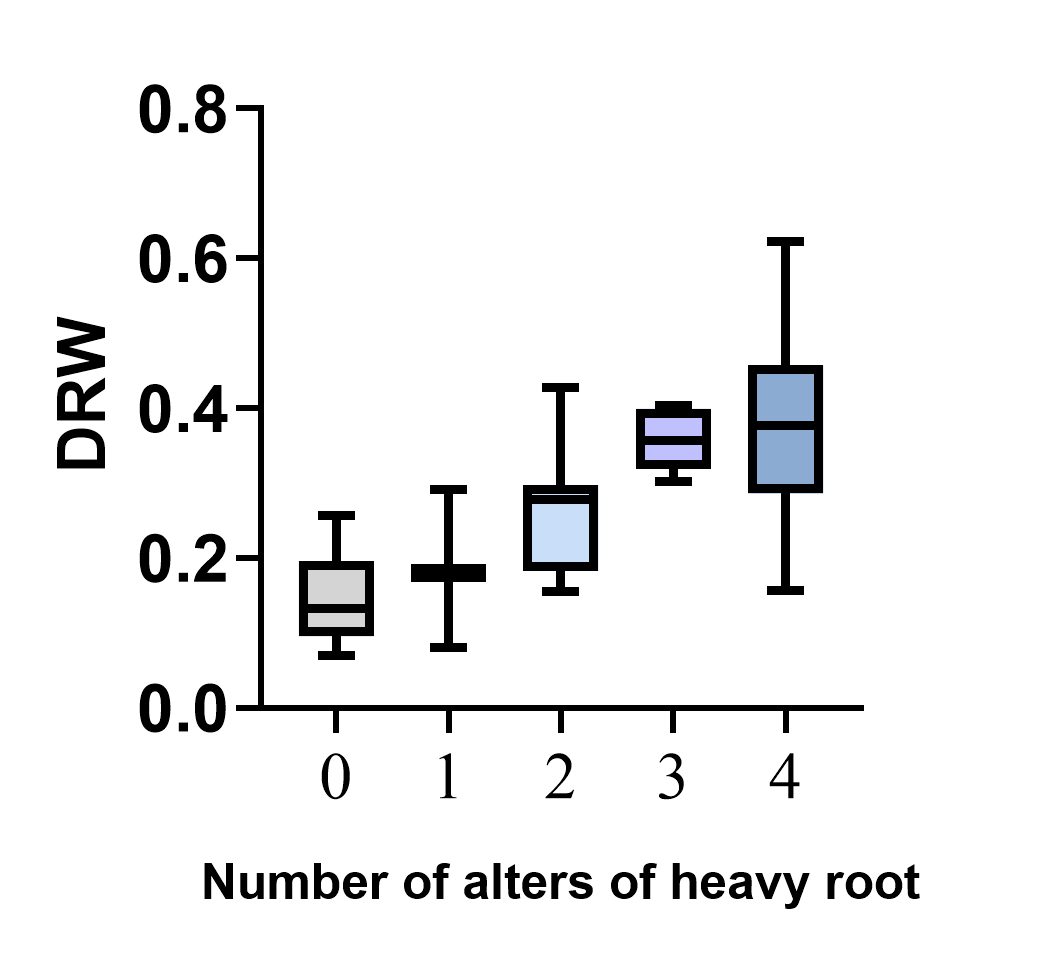


**Figure S5. Additive effect genetic analysis of GWAS locus.**

**
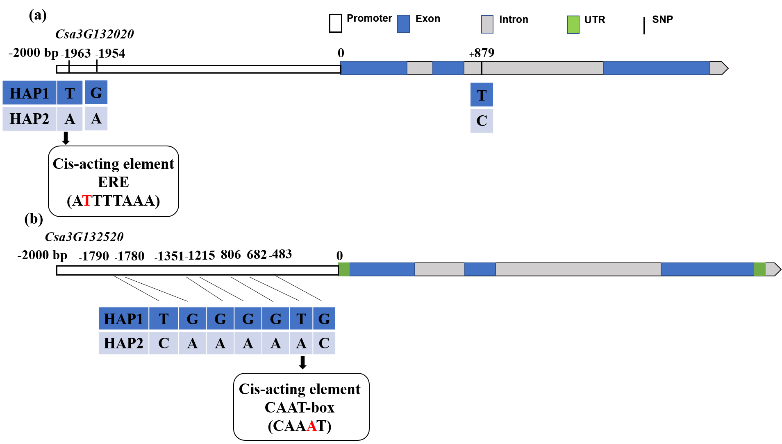
**

**Figure S6. Analysis of the cis-acting elements in the promoter of a candidate gene at the *gDRW3.1* locus.**

**
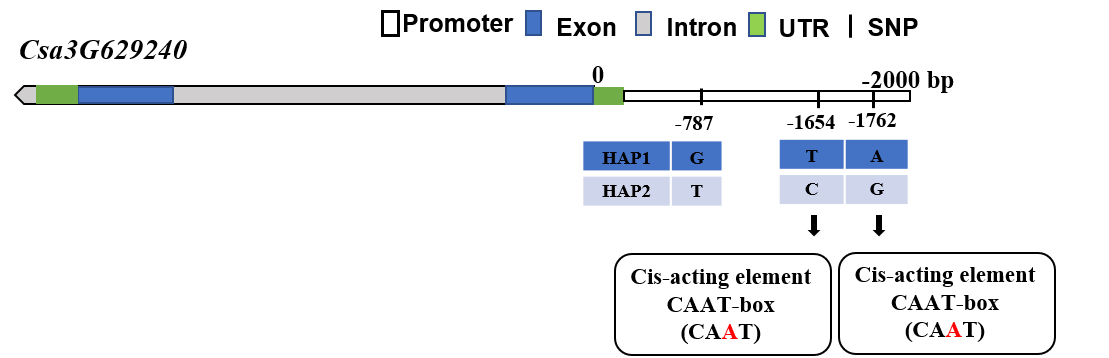
**

**Figure S7. Analysis of the cis-acting elements in the promoter of a candidate gene at the *gDRW3.2* locus.**

**
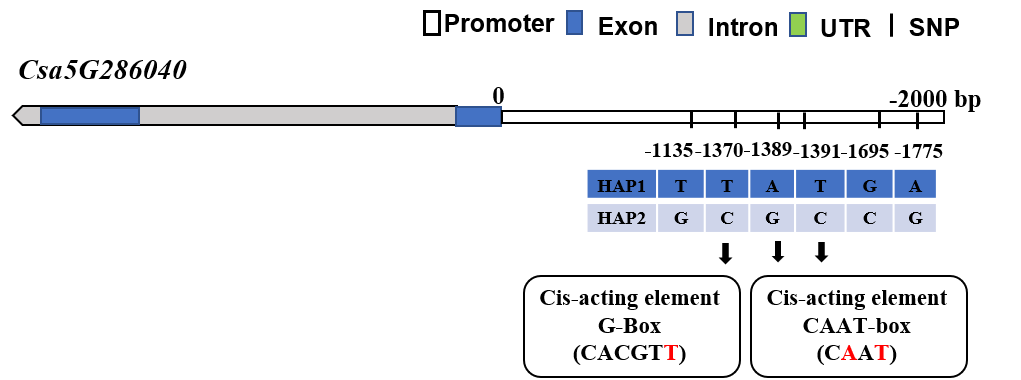
**

**Figure S8.** **Analysis of the cis-acting elements in the promoter of a candidate gene at the *gDRW5.1* locus.**
